# Machine learning and deep learning–based prediction of hypertension and analysis of its major risk factors in Bangladesh

**DOI:** 10.1371/journal.pone.0358471

**Published:** 2026-09-17

**Authors:** Shawrab Chandra, Md. Maeen Molla, Samiul Islam, Md. Matiur Rahaman, Mohammad Ali, Md. Ayub Ali

**Affiliations:** 1 Department of Statistics and Data Science, University of Barishal, Barishal, Bangladesh; 2 Department of Statistics, Pirojpur Science and Technology University, Pirojpur, Bangladesh; 3 Department of Statistics and Data Science, University of Rajshahi, Rajshahi, Bangladesh; 4 Department of Statistics, Faculty of Science, Gopalganj Science and Technology University, Gopalganj, Bangladesh; 5 Statistics Discipline, Khulna University, Khulna, Bangladesh; University of Porto Faculty of Medicine: Universidade do Porto Faculdade de Medicina, PORTUGAL

## Abstract

**Background:**

Hypertension is a leading cause of cardiovascular morbidity and mortality in Bangladesh. This study examined its prevalence, risk factors, and predictive modeling using machine learning (ML) and deep learning (DL) approaches.

**Method:**

We analyzed cross-sectional data from the 2022 Bangladesh Demographic and Health Survey, which included 14,283 adults (≥18 years). Prevalence was estimated, chi-square tests assessed associations, and four ML models (weighted logistic regression, random forest, extreme gradient boosting, light gradient boosting machine) and two DL models (TabNet, and multi-layer perceptron) were applied to predict hypertension risk. Model performance was evaluated using accuracy, precision, recall, specificity, F1 score, and area under the receiver operating characteristics curve and precision-recall curve.

**Results:**

Overall prevalence was 18.04% (95% CI: 17.2%–18.9%), higher among women (18.87%) than men (16.97%). The chi-square test suggests that hypertension was significantly associated with age, BMI, diabetes, wealth index, education, household size, and region (p < 0.05). Among the machine learning and deep learning models, weighted logistic regression (WLR) achieved the highest accuracy (0.817), precision (0.444), specificity (0.981), AUC-ROC (0.751), and AUC-PR (0.357). However, WLR exhibited low recall (0.070). In contrast, the random forest (RF) model achieved the highest recall (0.687) and F1-score (0.460) on the test data, indicating greater sensitivity in identifying individuals with hypertension. Additionally, age, BMI, sex, family size, and educational level were identified as the most important predictors among the variables included in the study.

**Conclusion:**

Hypertension is common in Bangladesh, with higher prevalence in women and significant association with socio-demographic determinants. Although WLR demonstrated the highest accuracy, precision, specificity, and AUC-PR, its low recall limits its utility for identifying individuals with hypertension. RF may be more suitable for public health applications because of its higher recall and F1-score; however, further external validation and assessment of its clinical utility are required before implementation.

## Introduction

Hypertension, commonly referred to as high blood pressure, it is a physical condition when the systolic blood pressure (SBP) ≥ 140 mmHg and/or diastolic blood pressure (DBP) ≥ 90 mmHg [1]. It is a major modifiable risk factor for cardiovascular diseases (CVDs), the leading cause of premature death [2]. Hypertension significantly increases the risk of various diseases [3], including heart attack [4], heart failure [5], coronary heart disease (CHD) [6], stroke [7], chronic kidney disease (CKD) [8], and diabetes [9]. Globally, hypertension affects an estimated 1.28 billion adults aged 30–79 years, two-thirds of whom live in LMICs [10]. By 2025, this figure is projected to reach 1.56 billion, driven by demographic changes, urbanization, dietary transitions, and sedentary lifestyles [11–13]. In 2019, CVDs caused about 18.6 million deaths, with nearly 80% occurring in low and middle-income countries (LMICs) [14].

While hypertension is a major global health concern, its impact is particularly alarming in Bangladesh, where 22.8 million adults aged 30–79 years have high blood pressure and more than 84% do not have their condition under control [15]. In Bangladesh, 14 of the 20 deaths were due to noncommunicable diseases, and the three leading causes were stroke, ischaemic heart disease, and chronic obstructive pulmonary disease [16]. According to the World Health Organization, approximately 283,000 people die from cardiovascular diseases in Bangladesh each year, of which 52% are linked to hypertension [15]. Furthermore, the expected annual cost of the hypertension control program was $3.2 million USD in Bangladesh [17].

In addition, increased life expectancy from improved healthcare has contributed to a growing burden of non-communicable diseases (NCDs) such as hypertension, whose prevalence is rising due to demographic, socioeconomic, and lifestyle factors [11,18,19]. Sex differences are evident, with men more prone earlier in life while postmenopausal women at greater risk due to reduced estrogen’s vasoprotective effects [20]. Metabolic factors, including elevated blood glucose and diabetes, share pathophysiological mechanisms with hypertension such as insulin resistance and vascular remodeling [21]. Socioeconomic status influences risk through healthcare access, diet quality, and stress exposure [22]. Environmental factors, including urban versus rural residence and regional differences, further influence hypertension prevalence; urbanization is associated with lifestyle patterns that promote hypertension [23].

Hypertension has been the focus of numerous studies [24,25], with a number of investigations conducted in Bangladesh using BDHS data [26–28]. For instance, Chowdhury et al., examined prevalence and risk factors among adults aged ≥35 years using logistic regression [29]. Iqbal et al., analyzed demographic, socioeconomic, and biological correlates, along with awareness, treatment, and control, using multivariate logistic regression [18]. Chowdhury et al., compared BDHS 2011 and 2017–18 data to assess changes in prevalence and risk factors, employing logistic regression and Wagstaff decomposition [19]. Sathi et al., explored trends in hypertension, diabetes, and their coexistence using modified Poisson regression [28]. Ghosh et al., estimated age-standardized prevalence and assessed determinants via hierarchical mixed-effects sequential Poisson regression [30]. Islam et al., applied feature selection (LASSO, SVM-RFE) and machine learning models (ANN, DT, RF, GB) to predict hypertension, with SVM-RFE combined with GB achieving the best performance [31].

Although machine learning methods are commonly used for hypertension prediction, applications of deep learning remain limited. In recent years, however, deep learning algorithms have been widely applied to predict a variety of diseases and have often outperformed traditional approaches. In this research, we employ both machine learning and deep learning techniques to predict hypertension using BDHS data, with the objective of identifying the most effective predictive model and the key risk factors associated with hypertension. We also investigated the prevalence of hypertension among adults’ people in Bangladesh.

## Materials and methods

### Study design, setting, participants, and study size

**Fig 1:**
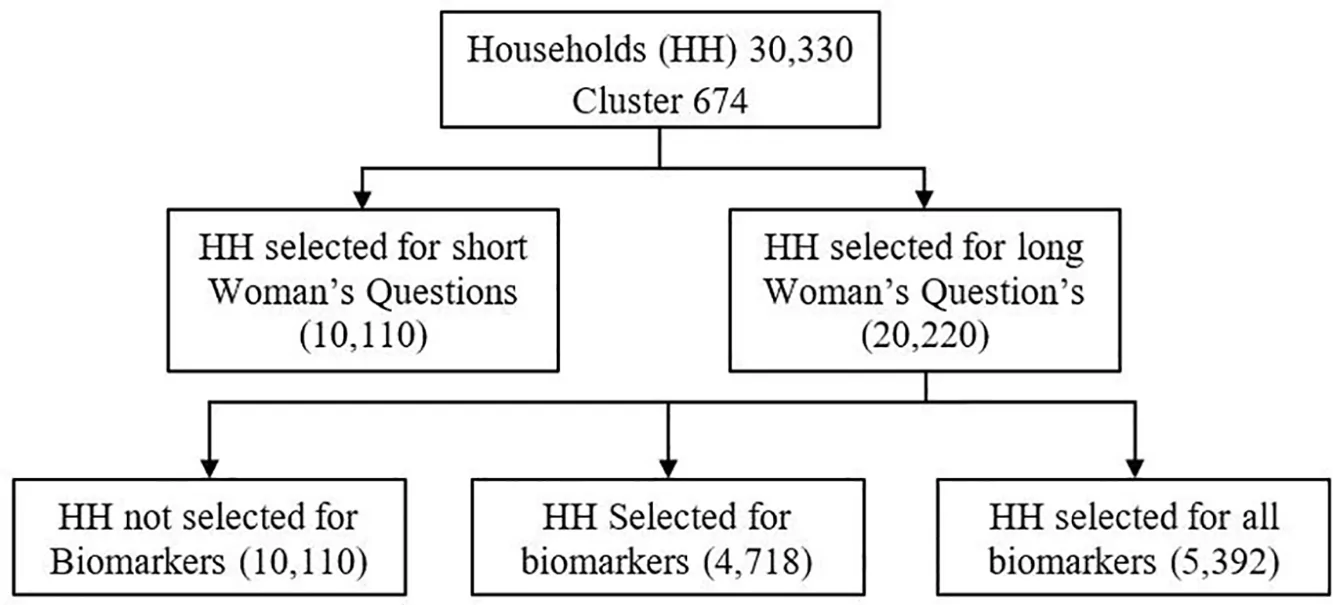
BDHS 2022 sample selection procedure.

Data for this study were obtained from the cross-sectional study of the Bangladesh Demographic and Health Survey (BDHS) 2022 [32]. Primary data were collected from June 27, 2022, to December 12, 2022, through face-to-face interviews, and biomarker measurements were taken by trained enumerators. BDHS 2022 was the third survey to include biomarker variables. It employed a two-stage stratified sampling design based on the integrated multi-purpose sampling master frame from a complete list of enumeration areas (EAs) covering the whole country. In the first stage, 674 EAs were selected using probability proportional to size; in the second stage, 45 households were randomly chosen per EA. Among 45 households, 30 households received the long questionnaire, where 15 were systematically selected for biomarker measurements, especially the women age between 15 and 49 and children under 5. Within this subsample, half of the households (8 of 15) were systematically selected for biomarker measurements among men aged ≥18 years, ever-married women aged ≥50 years, and never-married women aged ≥18 years, and blood pressure and blood glucose were measured for all men and women aged ≥18 years. Fig 1 shows that the 2022 BDHS included 30,330 households from 674 clusters, of which 20,220 were selected for the long women’s questionnaire and 5,392 for biomarker measurements. A total of 14,283 complete samples were obtained from 5,392 households, with no missing values across study variables.

### Ethical approval and consent to participates

In this study, we used publicly available secondary data from the Bangladesh Demographic and Health Survey (BDHS). The survey was conducted under the authority of the National Institute of Population Research and Training and the Medical Education and Family Welfare Division, Ministry of Health and Family Welfare. The procedures and questionnaires for the BDHS survey are approved by the ICF institutional review board (IRB) and Bangladesh IBR. In order to collect the information, an informed consent statement was given to the respondent, and the participation in the survey was voluntary. Also, the identification numbers of the respondents, such as the enumeration area, household number, and individual number, are destroyed and randomly reassigned. As a result, individuals or households are not identifiable.

### Response variable and explanatory variables

The variable of interest or response variable in this study is hypertension, commonly referred to as high blood pressure. Out of 30,330 households, 5,392 households were chosen as a measure of the biomarkers, and this measure includes the blood pressure. In which 6,853 men and 8,156 women aged 18 years or more could certainly undergo blood pressure and blood glucose level checkup. Blood pressure was measured in ninety-five percent of the female and ninety one percent of the male qualifiers. The respondents were measured three times each on blood pressure and the mean of the second and the third reading was taken. Participants were considered hypertensive if they SBP ≥ 140 mmHg or DBP ≥ 90 mmHg or were taking antihypertensive medication at that time to control it.

We selected 10 explanatory variables, including age, sex, marital status, family size, division, residence type, wealth index, education level, body mass index (BMI), and diabetes status, which have also been used in previous studies [11,18,28,29,31]. The list of selected explanatory variables and their categories is provided in Table 1. The BMI variable categorizes the participants as underweight (BMI < 18.5 kg/m^2^), normal (18.5 ≤ BMI < 25 kg/ m^2^) and obese/overweight (BMI ≥ 25 kg/ m^2^) group. Diabetes status was determined based on plasma blood glucose levels (mmol/L); a person was considered diabetic if their glucose level exceeded 7 mmol/L or if they were taking medication for the condition, otherwise they were classified as non-diabetic [33]. The variable family size is determined by the members of the family: small family has members less than 5, medium family has 5–7 members, and large family has more than 7 members.

**Table 1:** Description of the response and explanatory variable(s).

Variables	Categories
Hypertension	Yes; No
Age	18-34 years; 35–59 years; 60 + years
Sex	Male; Female
Marital status	Never married; Married
Family size	Small; Medium; Large
Wealth index	Poorest; Poorer; Middle; Richer; Richest
Education level	No education/primary education; Secondar/higher education
Division	Barisal; Chittagong; Dhaka; Khulna; Mymensingh; Rajshahi; Rangpur; Sylhet
Residence type	Urban; Rural
BMI	Underweight; Normal; Obese/Overweight
Diabetes status	Yes; No

### Statistical analysis

The baseline characteristics of the survey are indicated by frequency (%), at each variable. Also, the prevalence of hypertension is calculated by category with the help of the confidence interval (CI). Multicollinearity was assessed using the variance inflation factor (VIF). The value less than 5 indicates no major multicollinearity. The chi-square test was applied in determining the significance of the variables and 0.05 value of the significance level would be considered. Additionally, six machine learning and deep learning models were used to identify the risk factors of hypertension. For statistical analysis, we use R version 4.5.1. To incorporate the complex survey design, we use the survey package and svydesign functions in R programming.

### Dataset pre-processing

#### Handling the class imbalance of data.

Class imbalance occurs when samples of one class are significantly higher than the other class. A typical dataset is considered imbalanced if the majority class observations are twice or more than the minority class [34]. In our study, the response variable, hypertension, was divided into two categories: yes and no. The minority class (yes) consists of 18.04% of the data, while the majority class (no) consists of 81.96% of the data. Therefore, our dataset is imbalanced. We apply the synthetic minority oversampling technique (SMOTE) to address the class imbalance. SMOTE is applied only for the training data, not for test data. SMOTE was used for all the models except the weighted logistic regression model.

#### Missing data and categorical variables.

No imputation technique was used to handle missing data; we simply deleted the entire row from the dataset. The hypertension variable was encoded as a binary class (Yes = 1, No = 0), while other categorical predictors (sex, age group, family size, marital status, division, residence, wealth index, education, BMI, and diabetes) were encoded using one-hot encoding. To optimize scale-sensitive models such as MLP, features were standardized to zero mean and unit variance using StandardScaler, whereas no scaling was applied to other models (Weighted Logistic Regression, Random Forest, XGBoost, LightGBM, TabNet).

#### Train-test split.

We divided our dataset into two portion that is training and test data. For the training data, we kept 80% of it, and for the test, we kept the remaining 20%. During the train test split we stratified the data by the hypertension status. The 80/20 split was not explicitly constrained by survey cluster, observations from the same cluster could potentially occur in both the training and test sets. To assess the potential influence of cluster-level dependence, we additionally performed 5-fold and 10-fold StratifiedGroupKFold cross-validation, using survey cluster as the grouping variable.

### ML and DL models and parameter tuning

In our study we have used four machine learning (ML) and two deep learning (DL) models, including weighted logistic regression, random forest, eXtreme Gradient Boosting, Light Gradient Boosting Machine, TabNet, and multi-layer perceptron (MLP). In this study, the DHS sampling weights were used only in the weighted logistic regression, which is the baseline survey-weighted regression model. DHS sampling weights were not used in the training of the random forest, XGBoost, LightGBM, MLP, and TabNet models. We made this distinction because the purpose of the analysis is to compare various predictive performance of various ML and DL algorithms with a survey-weighted regression model.

#### Weighted logistic regression.

Let, there be a set of independent variables $x=(x_{\text{1}},x_{\text{2}},\ldots ,\;x_{p})$ and a binary dependent variable y. Now we have a set of n independent observations, ${(x}_{i\text{1}},\;x_{i\text{2}},\;\ldots ,\;x_{ip},\;w_{i},\;y_{i})$ for i=1,  2, ..., n  where $x_{ip}$ are the value of corresponding $\left(x_{\text{1}},x_{\text{2}},\ldots ,\;x_{p}\right)$, $w_{i}$ is the corresponding weight, and $y_{i}$ is the value of $i^{th}$ observation. Then the logistic regression model is defined as follows:


$$\begin{array}{c} \begin{array}{c} \pi (x)=\frac{e^{\beta_{\text{0}}+\beta_{\text{1}}x_{\text{1}}+\beta_{\text{2}}x_{\text{2}}+\ldots +\beta_{p}x_{p}}}{\text{1}+\;e^{\beta_{\text{0}}+\beta_{\text{1}}x_{\text{1}}+\beta_{\text{2}}x_{\text{2}}+\ldots +\beta_{p}x_{p}}\;}\; \end{array} \end{array}$$ (1)

Where, $\beta_{\text{0}},\;\beta_{\text{1}},\;\ldots ,\;\beta_{p}$ are the regression coefficient. Then, the likelihood function of weighted logistic regression is defined as follow:


$$\begin{array}{c} (X,\;W,\;\beta )=\prod_{i=\text{1}}^{n}\pi {\left(x_{i\text{0}},\;x_{i\text{1}},\ldots ,\;x_{ip}\right)}^{w_{i}y_{i}}{\left[\text{1}-\pi \left(x_{i\text{0}},\;x_{i\text{1}},\ldots ,\;x_{ip}\right)\right]}^{w_{i}\left(\text{1}-y_{i}\right)}\;\; \end{array}$$ (2)

The weighted logistic regression is useful when the data is imbalanced. It can handle the imbalanced data without synthetic data generation or resampling techniques [35,36].

#### Random forest (RF).

Random Forest is an ensemble algorithm that sequentially builds a number of decision trees, and based on those trees makes a prediction more accurate and stable model [37].

For hyperparameters in the RF model, we use the GridSearchCV class in sklearn.model_selection module where in param_grid we use n_estimators = [20, 50, 100, 150, 200, 300] and obtained the best parameter n_estimators = 150, the other parameters were max_depth = 10, min_samples_leaf = 30, class_weight = “balanced”, and random_state = 42.

#### Light Gradient Boosting Machine (LightGBM).

LightGBM is an ensemble learning framework based on gradient boosting, which builds a strong predictive model by sequentially adding decision trees that minimize a loss function via gradient descent [38].

For hyperparameters in the LightGBM model, we use the GridSearchCV class in sklearn.model_selection module where in param_grid we use parameters num_leaves = [5, 20, 30, 50], learning_rate = [0.01, 0.05, 0.1, 0.2], and n_estimators: [20, 50, 100, 150, 200, 300] and obtained the best parameters learning_rate = 0.2 n_estimators = 200, and num_leaves = 30.

#### Extreme gradient boosting (XGBoost).

XGBoost is a powerful and fast implementation of the gradient boosting framework, optimized to work with less structured data (usually structured data such as a table), with incredible predictive performance. Theoretically, it is well suited to machine learning competitions and practical applications due to its robustness, regularization properties, and computational efficiency [39].

For hyperparameters in the LightGBM model, we use the GridSearchCV class in sklearn.model_selection module where in param_grid we use parameters max_depth = [3, 5, 7, 9], learning_rate = [0.01, 0.05, 0.1], n_estimators = [25, 50, 100, 200, 300], subsample = [0.5,0.7, 0.8, 0.9], and colsample_bytree = [0.7, 0.8, 0.9] and obtained the best parameters max_depth = 9, learning_rate = 0.05, n_estimators = 300, subsample = 0.8, and colsample_bytree = 0.7.

#### TabNet.

TabNet is a deep learning model that is tailor-made to tabular data to unite the capacity of neural networks with interpretability in the attention mechanism. In contrast to traditional neural networks which treat features differently, TabNet utilizes sequential attention to make decisions by attending to most relevant ones at each decision step, enhancing performance and providing explainability [40].

To define the TabNet model we use parameter n_d = 16, n_a = 16, n_steps = 4, gamma = 1.5, lambda_sparse = 1e-3, optimizer_params = dict(lr = 2e-2), and verbose = 1. During the fitting of the model, we use max_epochs = 100, patience = 20, and batch_size = 32.

#### Multi-layer perceptron (MLP).

Multi-Layer Perceptron (MLP) is a form of feedforward artificial Neural network which is commonly used in classification because it modelled the complex, non-linear association between the input and output [41]. An MLP is composed of input layer, multiple hidden layers, and output layer, with each neuron of a given layer setting connection with every neuron on the next layer. The backpropagation algorithm trains these networks to approximate functions by a weight adjustment and bias adjustment.

For the MLP model we use three hidden layers = [256, 128, 64], activation = ‘ReLU’, dropout = [0.4, 0.3, 0.3], regularization = L2(0.001), optimizer = Adam (lr = 0.0003), loss = ‘binary_crossentropy’, epochs = 100, batch_size = 64.

### Performance measure metrics

Accuracy is the widely used performance metric to evaluate the performance of a model. But it is inappropriate when we deal with imbalanced data. The reason is that high accuracy is achievable by predicting the majority class, whereas our focus can be on the minority class. That is why we need other evaluation metrics. In this case precision and recall focus on the minority class. Precision is appropriate when the false positive is in focus. On the other hand, recall is appropriate when false negative is in focus. Only precision or recall cannot describe the performance of the whole model. Because it is possible that the model exhibits excellent recall but terrible precision and vice versa. In order to incorporate both precision and recall into a single score, we use the F1 score. Other two useful performance metrics for imbalanced data are area under receiver operating characteristics curve (AUC-ROC) and area under precision-recall curve (AUC-PR). AUC-PR focuses on the minority class. On the other hand, AUC-ROC doesn’t have bias toward the models at the cost of the majority class. Therefore, in our study we incorporate a range of accuracy metrics, including accuracy, precision, recall, specificity, F1 score, AUC-ROC, and AUC-PR in order to perfectly explain the model. The formula and description of confusion matrix and performance metrics are given in Table 2.

**Table 2:** Confusion matrix and performance metrics in binary classification.

Component	Definition/ Formula	
**Confusion Matrix**		
– True Positive (TP)	Correctly predicted positive cases	
– False Negative (FN)	Actual positives incorrectly predicted as negative	
– False Positive (FP)	Actual negatives incorrectly predicted as positive	
– True Negative (TN)	Correctly predicted negative cases	
**Performance Metrics**	**Formula**	**Description**
**Accuracy**	$\frac{TP+TN}{TP+FN+FP+TN}$	Overall proportion of correctly classified instances [42,43].
**Precision**	$\frac{TP\;}{TP+FP}$	Fraction of true positives among all predicted positives [42,44].
**Recall/ TPR**	$\frac{TP}{TP+FN}$	Fraction of actual positives correctly identified [42,43]
**Specificity**	$\frac{TN}{FP+TN}$	Specificity is the proportion of actual negatives identified correctly.
**F1 Score**	$\frac{\text{2}*Precision*recall}{Precision+Recall}$	The F1 score is a single value considering both precision and recall. The higher value of the F1 score indicates the better performance of the model.
**AUC-ROC**	$TPR\;=\;\frac{TP\;}{TP+FN},\;$ $FPR\;=\frac{FP\;}{FP+TN}\;$	The ROC curve is the visualization of TPR and FPR. AUC-ROC is the single value between 0 and 1 and indicates the area under the ROC curve. The higher values indicate the better performance of a model [43,45].
AUC-PR	$Precision=\frac{TP\;}{TP+FP}$ $Recall=\frac{TP}{TP+FN}$	The PR curve is the plot of precision vs recall. AUC-PR is the single value between 0 and 1 that indicates the area under the PR curve.

TPR, True positive rate; FPR, False positive rate; ROC, Receiver operating curve; AUC, Area under curve; PR, Precision-recall.

#### K-fold cross-validation.

K-fold cross-validation is a widely used resampling technique for evaluating the performance of machine learning and deep learning models and exhibiting the robustness of the model. In this approach, the dataset is partitioned into k equal folds. In each iteration, k − 1 folds are used to train the model, while the remaining fold is used for evaluation. This process is repeated k times and each fold serves as the validation set exactly once. The performance metrics obtained from the k iterations are averaged to produce a single, reliable estimate of model performance.

Several variants of k-fold cross-validation exist. In this study, we employed stratified group k-fold cross-validation, using survey cluster as the grouping variable to ensure that observations from the same cluster were not distributed across different folds while maintaining the distribution of the outcome as far as possible. To ensure the robustness and stability of the results, both 5-fold and 10-fold cross-validation are applied.

#### Overfitting in machine learning and deep learning model.

A model that performs well in training data but poorly in test data is said to be overfitted [46]. Overfitting occurs when a model learns not only the pattern in the data but also the noise. The use of flexible machine learning models, high-dimensional covariates, and class imbalance in complex survey data, overfitting is a potential concern. To mitigate this risk, several safeguards were implemented. Data were split into training and testing sets using stratification by hypertension status, and model performance was evaluated on an independent test set. Class imbalance was addressed using SMOTE applied only to the training data to avoid information leakage. Model complexity was controlled through regularization and architectural constraints, including dropout, L2 regularization, batch normalization, and early stopping for neural networks, as well as depth, leaf size, subsampling, and regularization parameters for tree-based and boosting models. TabNet incorporated sparsity regularization and early stopping. Furthermore, the robustness of the models was verified using 5-fold and 10-fold cross-validation.

### Software and programming languages

In this study we use several programming languages and software environments. R version 4.5.1 was employed in carrying statistical analyses and pre-processing the initial data. Also, for the performing ML and DL algorithm we use Python version 3.11.0. All the coding in Python were performed using Google Colaboratory, a cloud-based platform that offers a Jupyter notebook environment with GPU-accelerated computation hosting power.

## Results

### Basic descriptive statistics and statistical analysis

Table 3 shows the prevalence of hypertension among adults in Bangladesh in relation to diverse socio-demographic and health-related factors. Prevalence estimates together with their 95% confidence interval (CI) and the Chi-square test was used to check the relationship between each variable and status of hypertension. Existing prevalence of hypertension was higher in the females (18.87%, 95% CI: 17.78–19.95) than that of the males (16.97%, 95% CI: 15.79–18.15) and the difference was significant (p = 0.006). The prevalence of hypertension was highly significant to age (p < 0.001) where the greatest prevalence existed among the 60 years and above age group (37.0%, 95% CI: 34.63–39.36) followed by 35–59 years (21.18%, 95% CI: 19.8–22.57) and 18–34 years had the least prevalence (5.99%, 95% CI: 5.29–6.69). Family size was also inversely relevant with smaller families experiencing prevalence level of 22.57 (95% CI: 20.74–24.39) than the medium and large families (17.13%, 95% CI: 16.05–18.21 and 14.96%, 95% CI: 13.14–16.77 respectively) (p < 0.001). The marital status had also significant correlation (p < 0.001) with married having high prevalence (19.54%, 95% CI: 18.55–20.54) than never-married individuals (5.11%, 95% CI: 3.87–6.35). In the case of BMI categories, the prevalence of individuals in the obese or overweight category was high about 27.13% (95% CI: 25.41–28.84), whereas the prevalence of individuals with normal weight was 14.97% (95% CI: 13.99–15.96) and the underweight individuals was 13.09 (95% CI: 11.51–14.68) (p < 0.001). There was also regional variance (p = 0.002) with highest prevalence recorded in Rajshahi (21.89%, 95% CI: 18.76–25.02) and Khulna (19.56%, 95% CI: 17.21–21.91) whereas the lowest value shows Mymensingh (14.43%, 95% CI: 12.16–16.7). The prevalence of urban residents was slightly higher (19.49%, 95% CI: 17.82–21.17) compared to the prevalence of the rural residents (17.47%; 95% CI: 16.39–18.55) and was significantly different (p = 0.043). The trend of wealth index was positively graded (p < 0.001), and prevalence of hypertension was lower in the poorest quarter (14.18%, 95% CI: 12.57–15.78) that of the richest quintile (23.02%, 95% CI: 20.86–25.18). Level of education was also significantly associated with hypertension (p < 0.001) with prevalence in those with no or primary education 21.55% (95% CI: 20.24–22.85) compared with those with secondary or higher education of 14.25% (95% CI: 13.18–15.32). Hypertension also had a strong relationship with diabetes (p < 0.001) and the prevalence was significantly higher in patients having diabetes (24.66%, 95% CI: 22.52–26.79) than in patients without diabetes (16.62%, 95% CI: 15.69–17.54).

**Table 3:** Hypertension prevalence table.

Variables	Levels	Total (%)	Prevalence % (CI)	P-value ($\chi^{2})$
	**Total**	14,283 (100)	18.04	
**Sex**	**Male**	6387 (44.7)	16.97 (15.79–18.15)	0.006
	**Female**	7896 (55.3)	18.87 (17.78–19.95)	
**Age groups**	**18–34**	5542 (38.7)	5.99 (5.29–6.69)	<0.001
	**35–59**	6294 (44.1)	21.18 (19.8–22.57)	
	**60+**	2447 (17.2)	37 (34.63–39.36)	
**Family Size**	**Small**	3271 (23.8)	22.57 (20.74–24.39)	<0.001
	**Medium**	8187 (57.6)	17.13 (16.05–18.21)	
	**Large**	2825 (18.6)	14.96 (13.14–16.77)	
**Marital Status**	**Never married**	1611 (10.6)	5.11 (3.87–6.35)	<0.001
	**Married**	12672 (89.4)	19.54 (18.55–20.54)	
**Education Level**	**No/Primary**	7236 (51.6)	21.55 (20.24–22.85)	<0.001
	**Secondary/Higher**	7047 (48.4)	14.25 (13.18–15.32)	
**Division**	**Barisal**	1521 (6.2)	15.96 (14.38–17.55)	0.002
	**Chittagong**	2003 (17.9)	17.76 (15.39–20.12)	
	**Dhaka**	1927 (23)	17.3 (15.26–19.35)	
	**Khulna**	1848 (12.2)	19.56 (17.21–21.91)	
	**Mymensingh**	1634 (8.3)	14.43 (12.16–16.7)	
	**Rajshahi**	1829 (13.6)	21.89 (18.76–25.02)	
	**Rangpur**	1774 (12)	18.91 (16.58–21.25)	
	**Sylhet**	1747 (6.8)	15.3 (12.77–17.82)	
**Residence type**	**Urban**	4917 (27)	19.49 (17.82–21.17)	0.043
	**Rural**	9366 (73)	17.47 (16.39–18.55)	
**Wealth Index**	**Poorest**	2575 (18.1)	14.18 (12.57–15.78)	<0.001
	**Poorer**	2782 (20.5)	16.69 (14.93–18.46)	
	**Middle**	2719 (20)	16.93 (15.35–18.5)	
	**Richer**	2984 (20.9)	18.76 (17–20.53)	
	**Richest**	3223 (20.6)	23.02 (20.86–25.18)	
**BMI Category**	**Underweight**	2251 (15.4)	13.09 (11.51–14.68)	<0.001
	**Normal**	8114 (57.2)	14.97 (13.99–15.96)	
	**Obese/Overweight**	3918 (27.4)	27.13 (25.41–28.84)	
**Diabetes Status**	**No**	11788 (82.6)	16.62 (15.69–17.54)	<0.001
	**Yes**	2495 (17.4)	24.66 (22.52–26.79)	

### Performance evaluation of the ML and DL models

#### Comparison of the evaluation metrics of the ML and DL models.

The performance of machine learning models was evaluated using several evaluation metrics such as, accuracy, precision, recall, specificity, F1-scores, AUC-ROC, AUC-PR values, along with figures including, receiver operating characteristics (ROC) curve, precision-recall curve. Table 4 compares the different performance metrics of six different ML and DL models on the training and test datasets. The TabNet model demonstrates excellent performance on the training data achieves the highest accuracy (0.896), recall (0.884), F1-score (0.894), AUC-ROC (0.968), and AUC-PR (0.972). However, this level of performance does not extend to the test dataset, which suggests potential overfitting. On the other hand, weighted logistic regression achieved the highest accuracy (0.817), precision (0.444), specificity (0.981), AUC-ROC (0.751), and AUC-PR (0.357) on the test data. However, its accuracy of 81.7% was slightly below the 81.96% majority-class rate. Furthermore, the model showed a very low recall of 0.070 and an F1-score of 0.121, indicating that it struggled to identify the minority class. Whereas the random forest model achieved the highest recall (0.687) and F1 (0.460) on the test dataset. LightGBM exhibits strong performance on the training dataset, with high recall (0.853), F1-score (0.808), AUC-ROC (0.886), and AUC-PR (0.893), indicating an effective ability to capture nonlinear relationships. However, its performance declines on the test dataset, particularly in precision (0.344) and recall (0.621), suggesting reduced generalization and sensitivity to unseen data. XGBoost achieves excellent training performance, attaining high accuracy (0.882), precision (0.900), recall (0.860), F1-score (0.880), and strong discrimination capability as reflected by its AUC-ROC (0.955) and AUC-PR (0.962). Despite this, the model experiences a substantial drop in recall (0.328) and F1-score (0.357) on the test dataset, indicating overfitting and a tendency to favor the majority class during prediction. The multilayer perceptron (MLP) model achieved highest precision (0.938) on the training data, and other metrics for training data are accuracy (0.872), recall (0.797), specificity (0.947), F1-score (0.861), and AUC-ROC (0.950). However, on the test dataset, recall decreases markedly to 0.186, resulting in a low F1-score (0.258).

**Table 4:** Evaluation metrics of ML and DL models.

Model	Dataset	Accuracy	Precision	Recall	Specificity	F1-score	AUC-ROC	AUC-PR
**WLR**	**Train**	0.820	0.505	0.078	**0.983**	0.135	0.748	0.371
	**Test**	**0.817**	**0.444**	0.070	**0.981**	0.121	**0.751**	**0.357**
**RF**	**Train**	0.758	0.733	0.814	0.703	0.771	0.758	0.689
	**Test**	0.709	0.345	**0.687**	0.713	**0.460**	0.700	0.294
**LightGBM**	**Train**	0.797	0.767	0.853	0.742	0.808	0.886	0.893
	**Test**	0.718	0.344	0.621	0.740	0.443	0.745	**0.357**
**XGBoost**	**Train**	0.882	0.900	0.860	0.904	0.880	0.955	0.962
	**Test**	0.787	0.392	0.328	0.888	0.357	0.722	0.337
**TabNet**	**Train**	**0.896**	0.905	**0.884**	0.907	**0.894**	**0.968**	**0.972**
	**Test**	0.770	0.348	0.317	0.869	0.331	0.691	0.312
**MLP**	**Train**	0.872	**0.938**	0.797	0.947	0.861	0.950	0.959
	**Test**	0.807	0.419	0.186	0.943	0.258	0.735	0.346

WLR, weighted logistic regression; RF, random forest; LightGBM, light gradient boosting machine; XGBoost, extreme gradient boosting machine; MLP, multi-layer perceptron.

**Fig 2:**
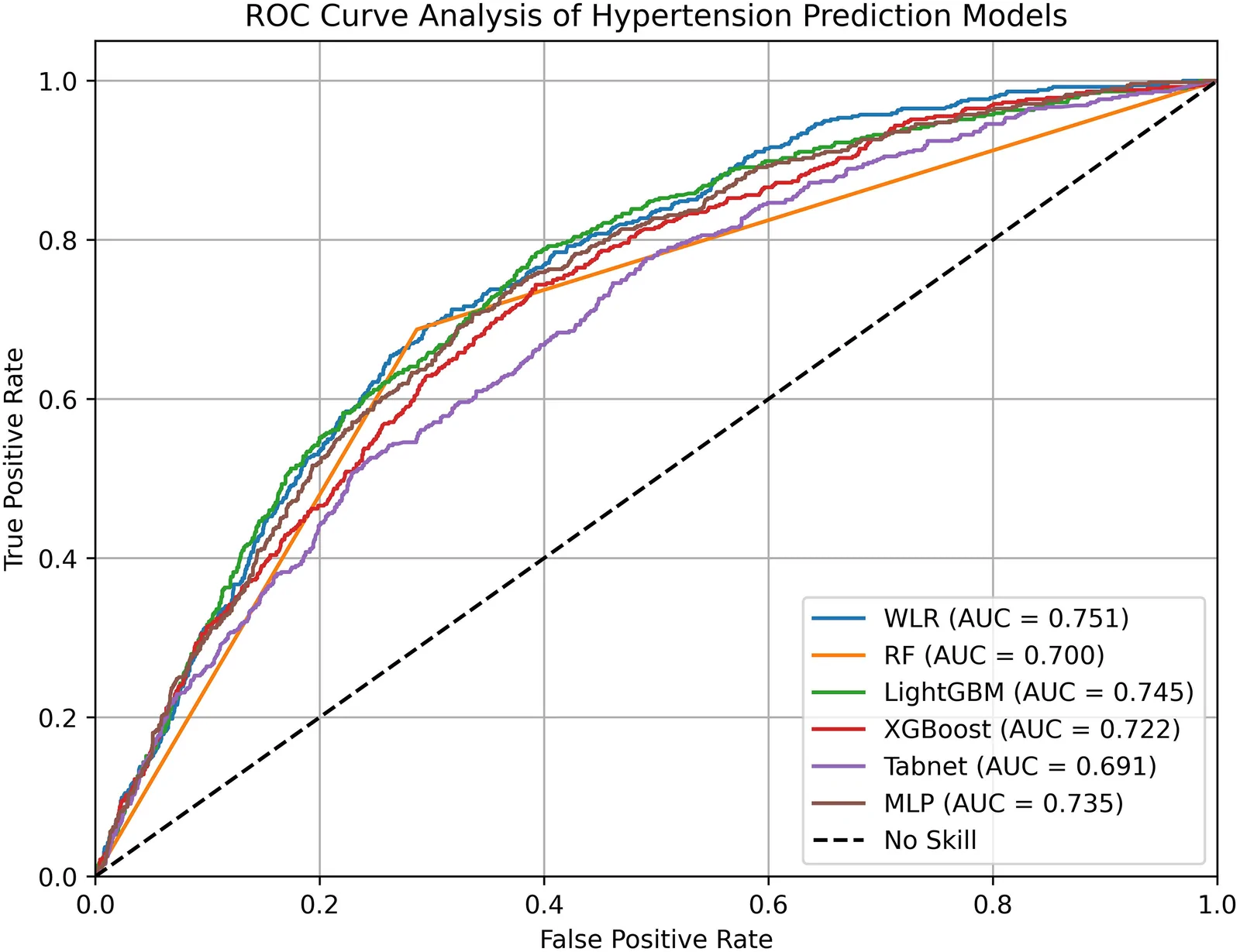
ROC curve of ML and DL models.

The receiver operating characteristics (ROC) curve of test data is presented in Fig 2. From Fig 2, we can see that WLR gained the highest area under the curve (AUC = 0.751), followed by LightGBM (AUC = 0.745) and MLP (AUC = 0.735), and random forest (AUC = 0.700), while TabNet recorded the lowest performance (AUC = 0.691).

**Fig 3:**
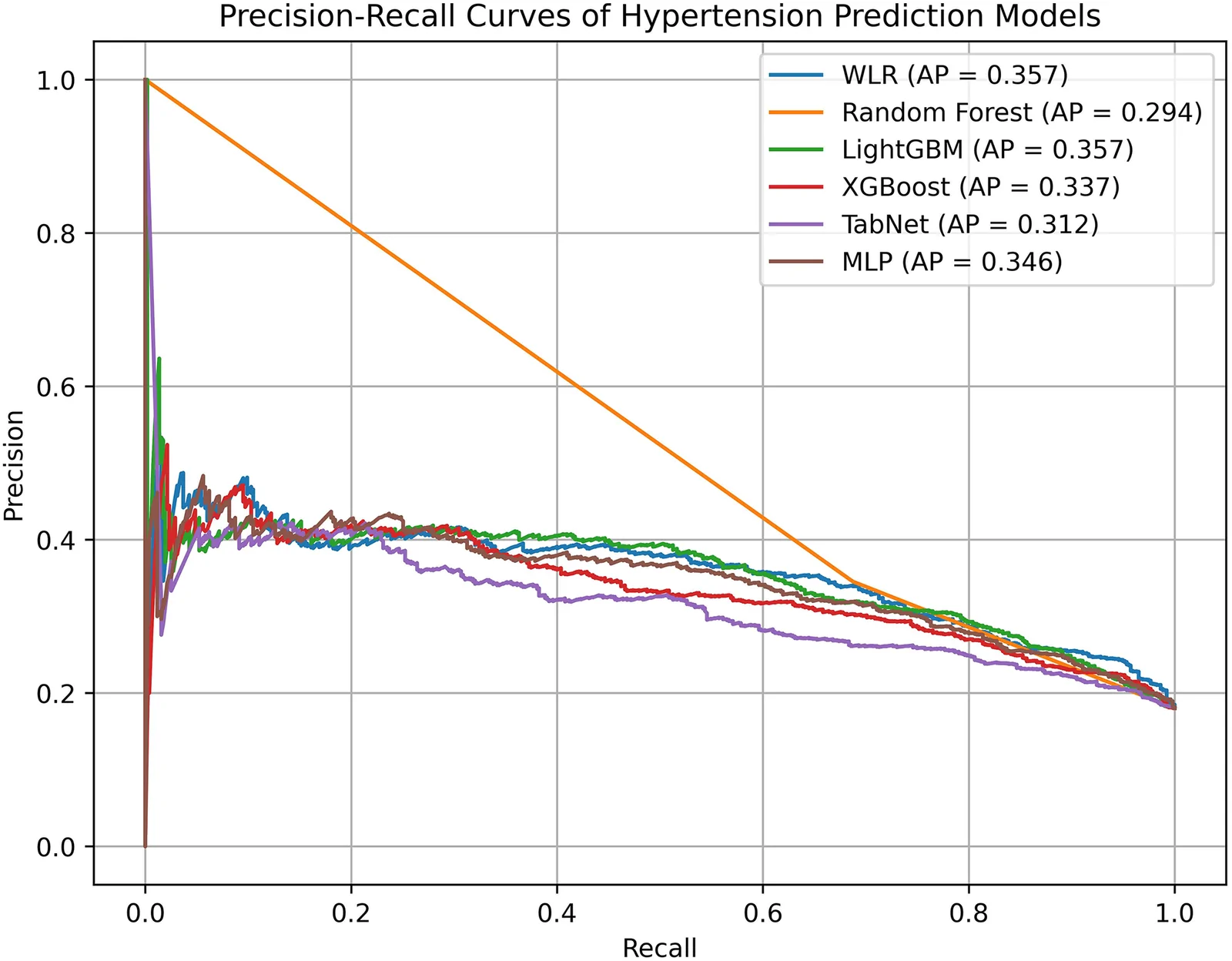
Precision-recall curve of ML and DL models.

Fig 3 displays the average precision that is equivalent to the area under the Precision–Recall (AUC-PR) curves. The value of the AUC-PR ranges from 0.294 to 357. Both WLR and LightGBM models achieved the same average precision with the value 0.357, which is the highest value. On the other hand, the random forest model gains the lowest average precision with a value of 0.294. Other models such as XGBoost, TabNet, and MLP models have the values 0.337, 0.312, and 0.346, respectively.

#### Cross-validation performance.

Table 5 summarizes the evaluation metrics of all models using 5-fold and 10-fold cross-validation (CV). Since the primary 80/20 train–test split was not cluster-disjoint, the cluster-based cross-validation results in Table 5 provide an additional robustness assessment of potential cluster-related performance inflation. Overall, the results show consistent performance across different values of *k*, with only minor variations in the mean estimates and standard deviations, indicating that the models are stable and not overly sensitive to the choice of folds. Overall, the cross-validation results highlight that no single model outperforms others across all evaluation metrics. The Weighted Logistic Regression (WLR) model achieved the highest mean accuracy (0.820), precision (0.503), AUC-PR (≈ 0.36) and specificity (≈ 0.98) for both 5-fold and 10-fold cross-validation. However, this strong performance on overall accuracy and specificity came at the cost of very low recall (≈ 0.08) and low F1-score (≈ 0.140), indicating that WLR performs poorly in identifying the minority (positive) class despite correctly classifying most negative cases. In contrast, the random forest (RF) model demonstrated the highest mean recall ≈ (0.67), AUC-ROC (0.748), and highest F1-score (≈ 0.44) across both values of *k*. This indicates that RF provides greater sensitivity to the positive class; however, its higher recall does not necessarily indicate superior overall discriminative ability.

**Table 5:** Performance metrices for the k-fold Cross-validation (k = 5 and 10).

Model	k	Mean Accuracy(±SD)	Mean Precision(±SD)	Mean Recall(±SD)	Mean Specificity(±SD)	Mean F1-score(±SD)	Mean AUC-ROC(±SD)	Mean AUC-PR(±SD)
**WLR**	**5**	**0.820** (±0.013)	**0.503** (±0.035)	0.081(±0.001)	**0.982** (±0.003)	0.140(±0.002)	0.747(±0.011)	**0.365** (±0.018)
	**10**	**0.820** (±0.007)	**0.503** (±0.072)	0.080(±0.016)	**0.983 (±0.004)**	0.138(±0.026)	0.745(±0.022)	**0.369** (±0.035)
**RF**	**5**	0.697 (±0.011)	0.332 (±0.021)	**0.670 (±0.020)**	0.703 (±0.014)	**0.443 (±0.017)**	**0.748 (±0.013)**	0.361 (±0.017)
	**10**	0.692 (±0.019)	0.328 (±0.024)	**0.675 (±0.028)**	0.695 (±0.021)	**0.442 (±0.026)**	**0.748 (±0.022)**	0.366 (±0.032)
**LightGBM**	**5**	0.720 (±0.010)	0.343 (±0.018)	0.603 (±0.019)	0.746 (±0.013)	0.436 (±0.011)	0.740 (±0.011)	0.350 (±0.015)
	**10**	0.715(±0.018)	0.339 (±0.026)	0.612 (±0.039)	0.737 (±0.020)	0.436 (±0.028)	0.738 (±0.022)	0.352 (±0.034)
**XGBoost**	**5**	0.773 (±0.006)	0.350 (±0.023)	0.305(±0.018)	0.876 (±0.006)	0.325 (±0.011)	0.719 (±0.010)	0.326 (±0.013)
	**10**	0.773 (±0.014)	0.359 (±0.042)	0.325 (±0.040)	0.872 (±0.016)	0.340 (±0.036)	0.718 (±0.022)	0.330 (±0.032)
**TabNet**	**5**	0.794(±0.015)	0.390(±0.030)	0.243(±0.058)	0.915(±0.026)	0.295(±0.037)	0.721(±0.010)	0.329(±0.013)
	**10**	0.787(±0.014)	0.368(±0.038)	0.243(±0.026)	0.907(±0.018)	0.292(±0.022)	0.721(±0.025)	0.324(±0.034)
**MLP**	**5**	0.793 (±0.012)	0.371 (±0.022)	0.211 (±0.024)	0.922 (±0.005)	0.269 (±0.022)	0.714 (±0.008)	0.319 (±0.015)
	**10**	0.789 (±0.010)	0.363 (±0.044)	0.219 (±0.026)	0.915 (±0.012)	0.272 (±0.029)	0.711 (±0.022)	0.321 (±0.027)

WLR, weighted logistic regression; RF, random forest; LightGBM, light gradient boosting machine; XGBoost, extreme gradient boosting machine; MLP, multi-layer perceptron.

For the other four models, LightGBM, XGBoost, TabNet, and MLP, the accuracy and AUC–ROC values range between 0.70 and 0.80. The lowest mean accuracy is observed for the LightGBM model using 10-fold cross-validation (0.715), whereas the highest mean accuracy is achieved by the MLP model with 10-fold cross-validation (0.789). In contrast, the MLP model with 10-fold cross-validation yields the lowest mean AUC–ROC value (0.711), while the LightGBM model with 5-fold cross-validation attains the highest mean AUC–ROC value (0.740). The mean recall for the LightGBM model using 10-fold cross-validation is 0.612, approximately twice as high as the XGBoost model and nearly three times higher than the MLP model.

#### Feature importance.

**Fig 4:**
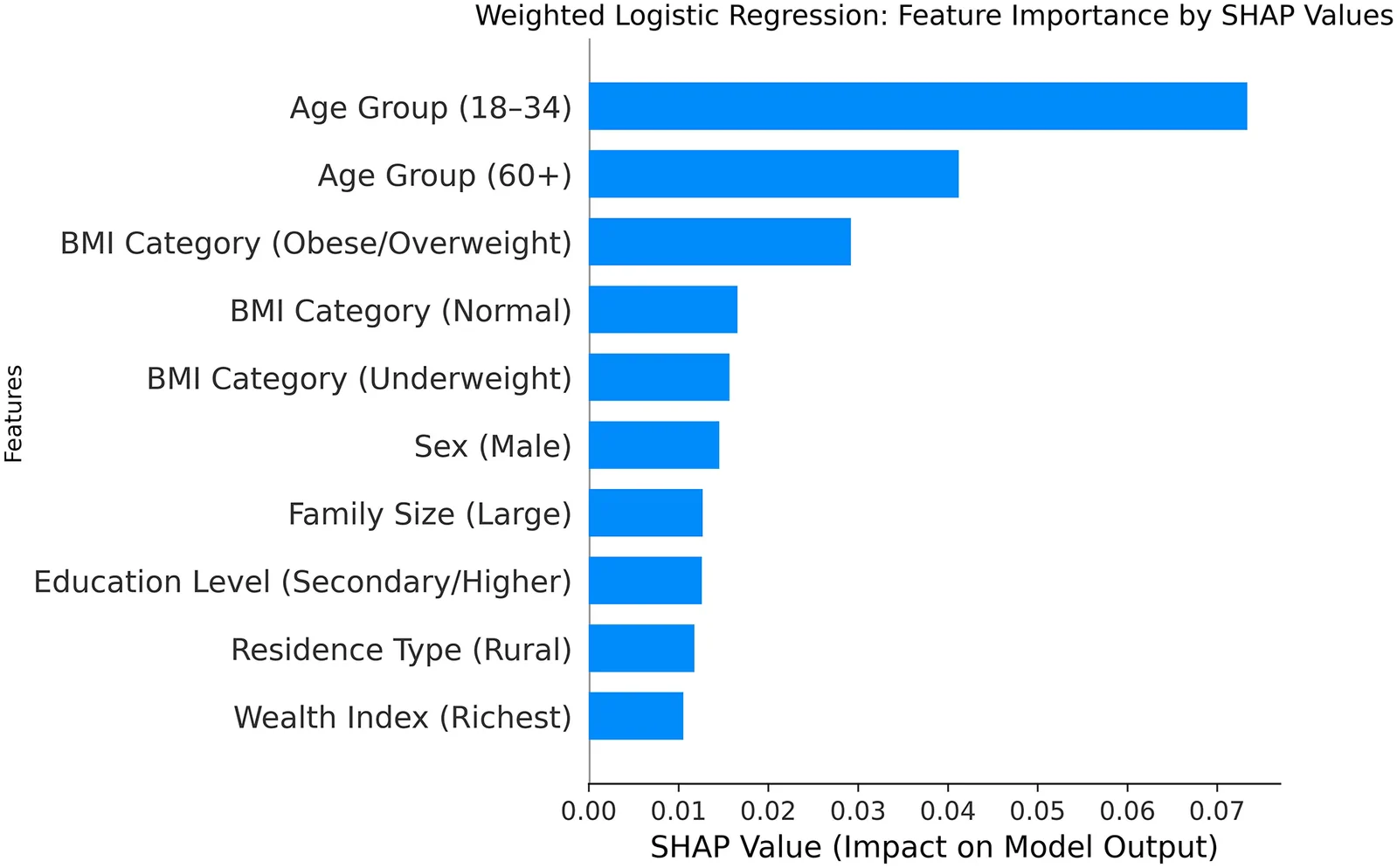
Top 10 most important features of weighted logistic regression model.

**Fig 5:**
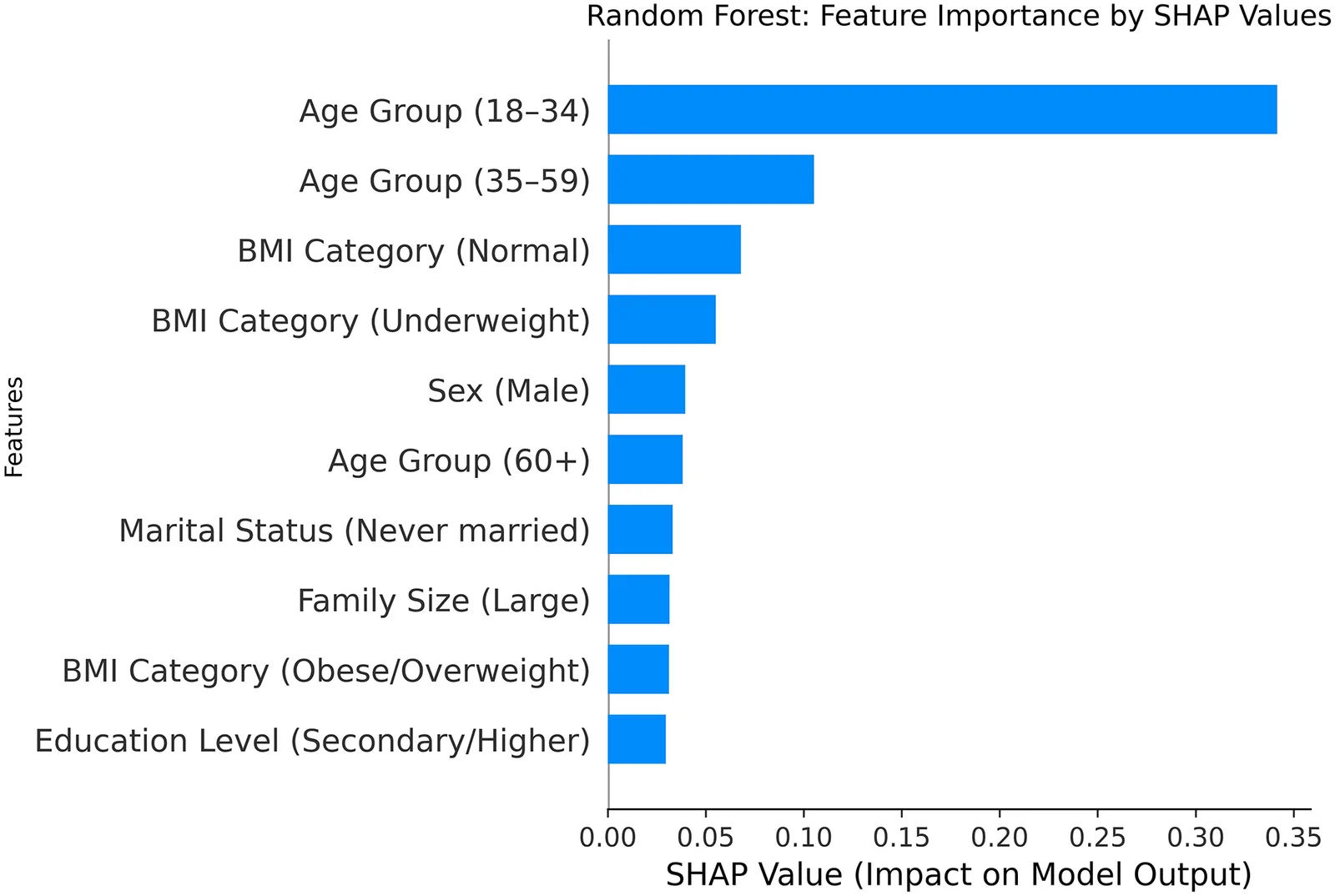
Ten most important features of random forest model.

Fig 4 and Fig 5 present the top ten features influencing the predicted probability of hypertension for the weighted logistic regression (WLR) and random forest (RF) models, respectively, based on SHAP values. As one-hot encoding was applied, each category of a categorical variable appears as a separate predictor in the models. Age and BMI are the most important factors for predicting hypertension for both the WLR and RF models. In WLR, the first two important variables are age group (18–34) and age group (60+), whereas in the RF model, age group (18–34) and age group (35–59) are the most important features, highlighting the strong role of age in hypertension prediction. After age, the variable BMI (obese/overweight, normal, underweight) was the significant factor in the WLR model, followed by sex (male), family size (large), education level (secondary/higher), residence type (rural), and wealth index (richest). Also, in the RF model, the BMI categories normal and underweight are found as significant predictors after the variable age group (35–59). Other significant variables in the RF model are sex (male), marital status (never married), family size (large), and education level (secondary/higher).

## Discussion

The paper explored the prevalence of hypertension in Bangladesh, predicted hypertension using machine learning (ML) and deep learning (DL) models, and identified the risk factors of hypertension. The results show that the overall prevalence of hypertension (18.04%) in the 2022 survey is lower than was in the 2011 and 2017–18 surveys [47,48]. We have explored six ML and DL algorithms—weighted logistic regression (WLR), random forest, LightGBM, XGBoost, TabNet, and multi-layer perceptron—to predict hypertension. Machine learning and deep learning models are used to predict different kinds of diseases, such as heart disease [49,50], thyroid disease [51], medical image classification tasks [49], and so on. Our dataset is imbalanced, and the minority class (presence of hypertension) consists of only 18.04% of the observations. Therefore, greater emphasis should be placed on evaluating model performance for this class. Some performance metrics are more sensitive to the minority class, such as precision, recall, F1-score, and the area under the precision–recall curve (AUC-PR), and are more appropriate than accuracy alone. The finding show that the WLR model provides the highest accuracy (0.817), precision (0.444), specificity (0.981), AUC-ROC (0.751), and AUC-PR (0.357) on test the dataset, while the random forest model provides the highest recall (0.687) and F1-score (0.460) on the test dataset. The findings of the 5-fold and 10-fold cluster-based cross-validation demonstrate identical performance with the test-set results, giving additional confirmation regarding the robustness of the findings in the presence of the clustered survey structure. Although the WLR model achieves high accuracy, specificity, AUC-ROC, and AUC-PR on the test dataset, its recall is extremely low (0.070), resulting in poor F1-scores (0.121). This indicates that WLR is heavily biased toward the negative class (no hypertension) and predicts more no hypertension when actually there is hypertension. In random forest, recall remains relatively high, but precision drops sharply (0.345), while the AUC-PR is (0.294). The higher recall should be interpreted in the context of the more permissive operating point associated with SMOTE-based class balancing and balanced weighting, which favors identification of the minority class but does not necessarily indicate overall better discriminative ability. Other models, including LightGBM, XGBoost, TabNet, and MLP, demonstrate good performance on the training data but fail to generalize to the test data, also indicating overfitting. In particular, the deep learning models TabNet and MLP achieved high values for the evaluation metrics on the training set. However, these values decrease approximately more than twofold on the test dataset, further confirming the presence of overfitting and limited generalization capability.

The age variable significantly influences the prediction of hypertension. The group of people age between 18 and 34 is the top contributor in both WLR and RF models, while different studies found that older people are more likely to develop hypertension [13,29,52,53]. These disparities can be explained by the phenomenon that our models perform better on the majority class (no hypertension), and in the age group (18–34), the prevalence of hypertension is 5.99% compared to the age group (35–59), 21.18%, and the age group (60+), 37%. Socioeconomic status and household living standards in Bangladesh have improved over time, which could lead to consuming unhealthy food and leading sedentary lifestyles, ultimately contributing to higher rates of obesity [54,55]. Also, consuming more calories than necessary might cause the body to retain them as fat, raise cholesterol, and cause hypertension [56]. BMI is the second most important predictor in predicting hypertension; previous study also found that the higher prevalence of hypertension is in the obese/overweight category [57,58]. Additionally, prevalence of hypertension in women is higher than in men, aligning with earlier studies [18,19], while sex (male) is an important predictor in both the WLR and RF models. Educational attainment also played significant role, with individuals secondary/higher education contributing more to hypertension prediction than individual with primary/no primary education. Previous studies have also reported that literacy level is significantly associated with hypertension [59,60].

The findings demonstrate the performance of ML and DL approaches in predicting hypertension using population-based survey data. While classical ML models demonstrate superior generalization than deep learning models in imbalanced situations, careful evaluation metrics selection and model interpretation are crucial to avoid misleading conclusions. Also, machine learning and deep learning models are exploratory in nature and are not intended for direct clinical screening or decision-making without further validation.

### Limitations of the study

This study has several limitations. First, the cross-sectional design of the BDHS data restricts causal inference, and longitudinal data would be more suitable for establishing causal relationships. Second, the dataset lacks important behavioral and clinical predictors, such as dietary salt intake, physical activity, alcohol consumption, family history of hypertension, and detailed dietary patterns, which may affect predictive performance. Third, there is a potential risk of algorithmic bias, particularly given existing socio-demographic and regional disparities, which may influence model fairness and generalizability across population subgroups. Fourth, DHS sampling weights were incorporated only into the WLR model and not into the other ML and DL models. Fifth, missing observations were excluded rather than imputed, which may have introduced selection bias. Although multiple strategies were employed to mitigate overfitting, it could not be completely eliminated, particularly in complex models and imbalanced data settings. Finally, model hyperparameters were largely based on default or commonly used values due to computational constraints; further tuning may improve performance in future studies.

## Conclusions

Using nationally representative data from BDHS, this study estimated the prevalence of hypertension and evaluated the performance of multiple machine learning (ML) and deep learning (DL) models for hypertension prediction. The overall prevalence of hypertension was 18.04%, with significant disparities across age, sex, education, wealth status, body mass index, diabetes status, residence type, and geographic region. Older age, overweight or obesity, diabetes, and socioeconomic factors were strongly associated with higher hypertension prevalence.

In predictive modeling, no single approach performed optimally across all evaluation metrics in this imbalanced dataset. Weighted logistic regression achieved the highest accuracy, specificity, and discrimination but exhibited very low recall and F1-score, limiting its utility for identifying hypertensive individuals. In contrast, the random forest model provided the highest recall and F1-score, indicating better detection of positive cases, although at the expense of precision and generalization. More complex models, including LightGBM, XGBoost, TabNet, and MLP, demonstrated substantial overfitting, with strong training performance but reduced test performance.

Feature importance analysis consistently identified age and body mass index as the dominant predictors of hypertension, followed by sex, education, household size, and socioeconomic status. Overall, the findings suggest that classical ML models may offer more reliable and interpretable performance than deep learning approaches for hypertension prediction using large, imbalanced population-based survey data. Recall, which represents the proportion of hypertensive patients correctly identified, was highest for the Random Forest model compared with the other models. Therefore, the Random Forest model may be more useful in a public health context. The findings of this study underscore the importance of model selection, appropriate evaluation metrics, and interpretability when applying ML methods to public health research. Future research using longitudinal data, enhanced feature sets, using other ML/DL methods, and advanced imbalance-handling techniques may further improve predictive performance and support evidence-based public health interventions.
