# Supplementary material for: Machine learning and deep learning–based prediction of hypertension and analysis of its major risk factors in Bangladesh
Source: PLoS One. 2026 Sep 17;21(9):e0358471. doi: 10.1371/journal.pone.0358471 (PMC13585235; doi:10.1371/journal.pone.0358471)
Supplement: Supplementary file 1 [file pone.0358471.s001.pdf]

Subject: Editorial Decision on Manuscript PONE-D-25-55897

Dear Authors,

Thank you for submitting your manuscript entitled **“Machine and deep learning–based prediction of hypertension and analysis of its major risk factors in Bangladesh”** to *PLOS ONE*. Your study addresses an important public health topic and applies contemporary machine learning and deep learning approaches to nationally representative data, which aligns well with the journal’s scope and multidisciplinary readership.

After careful editorial assessment of the manuscript, including evaluation of its scientific rigor, methodological transparency, and potential contribution to the literature, I am writing to inform you of the editorial decision:

Decision: Major Revisions Required

Below, I provide a detailed editorial summary outlining the main strengths of the manuscript, followed by the key issues that must be addressed before the manuscript can be considered for further evaluation.

1. Overall Assessment

The manuscript presents a well-conducted **cross-sectional analytical study** using data from the **2022 Bangladesh Demographic and Health Survey (BDHS)** to (i) estimate the prevalence of hypertension, (ii) examine associated socio-demographic and health-related factors, and (iii) develop predictive models using both machine learning (ML) and deep learning (DL) techniques.

The topic is **relevant and timely**, particularly for low- and middle-income countries facing a growing burden of noncommunicable diseases. The use of multiple ML/DL algorithms and comparison of their predictive performance represents a **methodologically sound and potentially valuable contribution**, although largely incremental relative to existing literature.

2. Key Strengths

- Use of a **large, nationally representative dataset** with appropriate handling of the complex survey design in descriptive and inferential analyses.
- Clear articulation of objectives and coherent structure across sections (Introduction, Methods, Results, Discussion).
- Application of multiple ML and DL models, with transparent reporting of core performance metrics (accuracy, precision, AUC).
- Appropriate acknowledgment of the main limitation related to the cross-sectional nature of the data.
- Relevance to public health surveillance and population-level risk stratification in resource-limited settings.

3. Major Issues Requiring Revision

The following points must be addressed comprehensively in a revised version of the manuscript:

3.1. Reporting Standards and Methodological Transparency

- The study design corresponds to an **observational cross-sectional study**, and therefore adherence to the **STROBE reporting guidelines** is expected.

- Please include a completed **STROBE checklist** as a supplementary file and ensure that all relevant items are adequately addressed in the manuscript.
- While the ML/DL modeling is described, key aspects related to **model robustness** require further clarification:
- Explicit discussion of potential **overfitting**.
- Clarification on whether any form of **cross-validation** (e.g., k-fold) was considered or why it was not applied.
- Stronger justification for the choice of performance metrics and thresholds, particularly given the class imbalance typical of hypertension prevalence data.

3.2. Interpretation of Predictive Performance

- The reported AUC values (approximately 0.73 at best) indicate **moderate discriminatory performance**.
- The Discussion and Conclusions currently risk **overstating the practical applicability** of the models.
- Please temper claims regarding the utility of DL models for real-world screening or decision-making, and clearly distinguish **statistical performance** from **clinical or policy relevance**.
- Many of the most important predictors (e.g., age, BMI, education) are well-established risk factors.
- Please clarify the **added value** of the ML/DL approach compared with traditional regression-based models beyond marginal gains in predictive accuracy.

3.3. Ethical Statement

- The Ethics Statement currently reports "N/A."
- Although the study uses publicly available, anonymized secondary data, *PLOS ONE* requires a **clear ethical justification**, including:
- Identification of the data source as publicly available.
- A statement that the analysis involved no identifiable human subjects and therefore did not require institutional ethical approval, or confirmation of exemption where applicable.

3.4. Reproducibility and Open Science

In line with *PLOS ONE*'s commitment to transparency and reproducibility:

- Please clarify whether the **analysis code (R/Python scripts)** can be shared, and if so, indicate how and where it will be made available (e.g., public repository).
- If code sharing is not possible, provide a clear justification.

3.5. Discussion of Bias and Limitations

The manuscript would benefit from a more explicit discussion of:

- **Residual confounding** and limitations inherent to self-reported or single-occasion measurements.
- Potential **algorithmic bias**, particularly given socio-demographic and regional disparities.
- The absence of behavioral variables (e.g., diet, physical activity, salt intake) and how this may affect predictive performance.

4. Minor but Important Points

Please ensure consistent terminology when referring to performance metrics (e.g., avoid interchangeably using “accuracy” and “precision” incorrectly).

Review the language in the Conclusions to ensure it remains fully supported by the results.

Carefully proofread the manuscript for minor grammatical and typographical issues.

5. Editorial Recommendation

The manuscript demonstrates **scientific merit and relevance**, but substantial revisions are required to strengthen methodological transparency, ethical reporting, and the interpretation of findings. Provided that the concerns outlined above are addressed thoroughly and convincingly, the manuscript may be suitable for reconsideration.

We invite you to submit a **revised manuscript** along with a **detailed point-by-point response** to each comment raised in this decision letter.

Thank you for your interest in *PLOS ONE*. We look forward to receiving your revised submission.

Sincerely,

Academic Editor
PLOS ONE
