# Supplementary material for: Machine learning and deep learning–based prediction of hypertension and analysis of its major risk factors in Bangladesh
Source: PLoS One. 2026 Sep 17;21(9):e0358471. doi: 10.1371/journal.pone.0358471 (PMC13585235; doi:10.1371/journal.pone.0358471)
Supplement: Supplementary file 2 [file pone.0358471.s002.docx]

**Response to the Editor and Reviewer**

Dear Editor and Reviewers,

We would like to extend our heartfelt appreciation to the academic editor and reviewers for generously devoting their time and offering insightful feedback on our manuscript titled "Machine and deep learning–based prediction of hypertension and analysis of its major risk factors in Bangladesh". We are grateful for the constructive comments provided, as they have enabled us to enhance the overall quality and coherence of our research. In light of the raised concerns, we have addressed each point in a comprehensive manner as outlined below:

**3.1. Reporting Standards and Methodological Transparency**

**Editor comment:** The study design corresponds to an observational cross-sectional study, and therefore adherence to the STROBE reporting guidelines is expected.

**Author’s response:** We thank the Editor for the helpful comments and suggestions. We have revised our manuscript and ensured full compliance with the STROBE reporting guidelines.

**Editor comment:** Please include a completed STROBE checklist as a supplementary file and ensure that all relevant items are adequately addressed in the manuscript.

**Author’s response:** We thank the Editor for this valuable suggestion. A completed STROBE checklist has now been included as supplementary file S1_file.pdf. All applicable STROBE items have been systematically addressed in the revised manuscript, including participant selection, variable definitions, handling of missing data, and statistical methods, and so on.

**Editor comment:** While the ML/DL modeling is described, key aspects related to model robustness require further clarification: Explicit discussion of potential overfitting.

**Author’s response:** We thank the Editor for this important comment. We have addressed potential overfitting in the subsection Overfitting in machine and deep learning model of Materials and Methods section (page: 11; lines: 232 - 243) and the Discussion (page: 18; lines: 370 - 375) sections.

**Editor comment:** Clarification on whether any form of cross-validation (e.g., k-fold) was considered or why it was not applied.

**Author’s response:** We thank the Editor for raising this point. We have used 5-fold and 10-fold cross-validation to validate our results. K-fold cross-validation has been explicitly discussed in the Materials and Methods section (page: 11; lines: 222 - 231) and in the Results section (page: 16; lines: 314 - 336).

**Editor comment:** Stronger justification for the choice of performance metrics and thresholds, particularly given the class imbalance typical of hypertension prevalence data.

**Author’s response:** We thank the Editor for this helpful suggestion. We have expanded the justification for the selected performance metrics in the Materials and Methods section (page: 10; lines: 206 - 221). We explain that metrics such as AUC-ROC, precision, recall, specificity, F1-score, and AUC-PR are more appropriate than accuracy alone for evaluating models trained on imbalanced outcome data such as hypertension prevalence.

**3.2. Interpretation of Predictive Performance**

**Editor comment:** The reported AUC values (approximately 0.73 at best) indicate moderate discriminatory performance.

**Author’s response:** We appreciate the Editor’s careful assessment. We agree with this assessment. We reanalyzed our data by applying SMOTE to address class imbalance, added a weighted logistic regression model, and expanded the set of performance metrics to include precision, recall, specificity, F1-score, AUC-PR, and AUC. Throughout the manuscript, we have avoided overstating the discriminatory performance of the model.

**Editor comment:** The Discussion and Conclusions currently risk overstating the practical applicability of the models.

**Author’s response:** We thank the Editor for this valuable observation. The Discussion and Conclusions have been revised to temper claims regarding practical applicability. The predictive performance is now described as moderate, and limitations related to real-world implementation are clearly acknowledged.

**Editor comment:** Please temper claims regarding the utility of DL models for real-world screening or decision-making, and clearly distinguish statistical performance from clinical or policy relevance.

**Author’s response:** We appreciate this important suggestion. We have clarified that the ML/DL models are exploratory in nature and are not intended for direct clinical screening or decision-making without further validation in the discussion section (page: 19; lines: 391 - 395).

**Editor comment:** Many of the most important predictors (e.g., age, BMI, education) are well-established risk factors.

**Author’s response:** We appreciate the Editor’s comment. We acknowledge that age, BMI, and education are well-established risk factors for hypertension. The discussion now explicitly situates these findings within the existing literature and cites previous studies to contextualize their importance in Discussion section (page:19; lines: 376 - 390).

**Editor comment:** Please clarify the added value of the ML/DL approach compared with traditional regression-based models beyond marginal gains in predictive accuracy.

**Author’s response:** We thank the Editor for this thoughtful comment. We have clarified that, in our study, the survey-weighted logistic regression model performed comparably or better on test data. The added value of ML/DL approaches is discussed in terms of their ability to capture nonlinear relationships rather than superior predictive accuracy, and claims of DL superiority have been avoided in the result and discussion section.

**3.3. Ethical Statement**

**Editor comment:** The Ethics Statement currently reports “N/A.”

**Author’s response:** We thank the Editor for highlighting this issue. We have revised the Ethics Statement to comply fully with PLOS ONE requirements

**Editor comment:** Although the study uses publicly available, anonymized secondary data, PLOS ONE requires a clear ethical justification, including:

Identification of the data source as publicly available.

A statement that the analysis involved no identifiable human subjects and therefore did not require institutional ethical approval, or confirmation of exemption where applicable.

**Author’s response:** We appreciate the Editor’s guidance. We have added a new sub-section titled 'Ethical Approval and Consent to Participate' (page 4: lines: 88–96). The revised statement clarifies that the study utilized publicly available, anonymized secondary data from the Bangladesh Demographic and Health Survey (BDHS). Since the research involved no identifiable human subjects, formal institutional ethical approval was not required.

**3.4. Reproducibility and Open Science**

**Editor comment:** In line with PLOS ONE’s commitment to transparency and reproducibility:

Please clarify whether the analysis code (R/Python scripts) can be shared, and if so, indicate how and where it will be made available (e.g., public repository).

If code sharing is not possible, provide a clear justification.

**Author’s response:** We thank the Editor for this important suggestion. We have created a new section titled “Data and Code Availability” (page: 5; lines: 97 - 102). This sub-section states that the analysis scripts used in this study are shared via a public repository, and the repository link has been provided.

**3.5. Discussion of Bias and Limitations**

**Editor comment:** The manuscript would benefit from a more explicit discussion of: Residual confounding and limitations inherent to self-reported or single-occasion measurements.

**Author’s response:** We thank the Editor for this valuable suggestion. We have created a new sub-section in the manuscript titled “Limitations of the Study” (page: 19; lines: 396 - 406). In this section, we note that the data are cross-sectional and self-reported, which may introduce residual confounding.

**Editor comment:** Potential algorithmic bias, particularly given socio-demographic and regional disparities.

**Author’s response:** We appreciate this insightful comment. In the sub-section “Limitations of the Study” (page: 19; lines: 396 - 406), we have added a discussion of potential algorithmic bias arising from socio-demographic and regional disparities.

**Editor comment:** The absence of behavioral variables (e.g., diet, physical activity, salt intake) and how this may affect predictive performance.

**Author’s response:** We thank the Editor for this helpful observation. In the sub-section “Limitations of the Study” (page: 19; lines: 396 - 406), we now explicitly acknowledge the absence of behavioral variables such as diet, physical activity, and salt intake as an important limitation and discuss how their exclusion may affect predictive performance.

**4. Minor but Important Points**

**Editor comment:** Please ensure consistent terminology when referring to performance metrics (e.g., avoid interchangeably using “accuracy” and “precision” incorrectly).

**Author’s response:** We thank the Editor for noting this issue. Throughout the manuscript, we have avoided the incorrect interchangeable use of terms such as accuracy and precision.

**Editor comment:** Review the language in the Conclusions to ensure it remains fully supported by the results.

**Author’s response:** We appreciate the Editor’s suggestion. The Conclusions have been revised to ensure that all statements are fully supported by the reported results and appropriately qualified.

**Editor comment:** Carefully proofread the manuscript for minor grammatical and typographical issues.

**Author’s response:** We thank the Editor for this suggestion. The manuscript has been carefully proofread, and minor grammatical and typographical issues have been corrected.

**Reviewer #1**: Address class imbalance explicitly (e.g., via class weights in MLP/TabNet or SMOTE). Report full metrics (accuracy, precision, recall, specificity, F1, AUC-ROC, AUC-PR) in a table for all models on both train and test sets. Emphasize F1 or AUC-PR over accuracy in imbalanced contexts.

**Author’s response:** We sincerely thank the Reviewer for his careful reading of the manuscript and his valuable comments. We have explicitly addressed class imbalance using SMOTE and provide performance metrics, including accuracy, precision, recall, specificity, F1-score, AUC-ROC, and AUC-PR for all models across both the training and test datasets. In the Materials and Methods section, we have added a sub-section on “Handling class imbalance” (page: 6; lines: 131 - 138) and updated the 'Performance Metrics' subsection (lines: 186 - 202) to further discuss the evaluation of imbalanced data.

**Reviewer #1**: Implement k-fold cross-validation (stratified by hypertension status and survey clusters) for robust performance estimates. Report mean/SD across folds.

**Author’s response:** We sincerely thank the Reviewer for this helpful suggestion. Stratified group k-fold cross-validation was used, with hypertension status guiding stratification and survey clusters treated as grouping units to prevent data leakage. We implemented 5-fold and 10-fold cross-validation and now report the mean values and standard deviations for all performance metrics across the folds. The K-fold cross-validation methodology is detailed in the Materials and Methods section (page: 10; lines: 206 - 221)., and the corresponding outcomes are presented in the Results section (page: 16; lines: 314 - 336).

**Reviewer #1**: Include a baseline model (e.g., survey-weighted logistic regression) for comparison. If DL outperforms, quantify (e.g., via McNemar's test).

**Author’s response:** We thank the Reviewer for this valuable suggestion. We have included a survey-weighted logistic regression (WLR) model as a baseline. The model is described in the Materials and Methods section (page: 7; lines: 153 - 163), and its findings are presented in the Results section.

**Reviewer #1**: Specify how feature importance was computed (e.g., for MLP, use SHAP instead of unspecified method). Provide uncertainty (e.g., via bootstrapping).

**Author’s response:** We appreciate the Reviewer’s suggestion. Feature importance was assessed using SHAP analysis, and the corresponding results are presented in the Results section (page 17; lines: 337 - 350).

**Reviewer #1**: Account for survey design in modeling (e.g., incorporate weights in loss functions or use survey-aware splits).

**Author’s response:** We thank the Reviewer for raising this important point. We have employed a stratified train–test split based on hypertension status to ensure that the class distribution was preserved across both subsets.

**Reviewer #1**: Tone down conclusions: Acknowledge modest AUC/F1 and limitations of cross-sectional data for causality. Avoid overclaiming DL "superiority" without baselines.

**Author’s response:** We sincerely thank the Reviewer for this helpful recommendation. We have toned down the Conclusions (page: 20; lines: 407 - 427), acknowledged the modest AUC and F1 values, acknowledged no superiority of a single model, and avoided claims of DL superiority.

**Reviewer #1**: Expand limitations: Discuss lack of behavioral variables (e.g., diet, exercise) and potential overfitting. Suggest future longitudinal validation.

**Author’s response:** We appreciate the Reviewer’s valuable suggestion. We have expanded the Limitations section (page: 19; lines: 396 - 406) to address the cross-sectional design of the study, the absence of behavioral data, potential risks of overfitting, and the need for longitudinal studies.

We've incorporated all feedback from the editor and reviewers, ensuring the revised manuscript aligns with PLOS ONE's high standards. We do believe the changes substantially enhance the research quality and contribution.

We reiterate our deep appreciation for their invaluable expertise and insights that greatly improved our work. We trust this revised version merits publication in PLOS ONE.

Thank you for your time and consideration.
