# Supplementary material for: Machine learning and deep learning–based prediction of hypertension and analysis of its major risk factors in Bangladesh
Source: PLoS One. 2026 Sep 17;21(9):e0358471. doi: 10.1371/journal.pone.0358471 (PMC13585235; doi:10.1371/journal.pone.0358471)
Supplement: Supplementary file 3 [file pone.0358471.s003.docx]

**Response to the Reviewers**

Dear Reviewers,

We would like to extend our heartfelt appreciation to the reviewers for generously devoting their time and offering insightful feedback on our manuscript titled "Machine and deep learning–based prediction of hypertension and analysis of its major risk factors in Bangladesh". We are grateful for the constructive comments provided, as they have enabled us to enhance the overall quality and coherence of our research. In light of the raised concerns, we have addressed each point in a comprehensive manner as outlined below:

**Reviewer #2’s Comments:** 1. Contextualization of Local Impact: While the introduction effectively outlines the global burden of hypertension, it lacks specific data regarding its impact within Bangladesh itself. The transition from the global context to the local scenario would be significantly stronger if the authors included local statistics on the consequences of the disease (e.g., mortality rates, morbidity, or specific economic burden in Bangladesh), rather than just stating that its prevalence is rising.

**Author’s response:** Thank you for this valuable suggestion. We agree that the original introduction did not sufficiently contextualize the burden of hypertension within Bangladesh. In response, we have expanded the introduction to include Bangladesh-specific evidence on the health and economic impact of hypertension (Page 3, Lines 48 - 54).

**Reviewer #2’s Comments:** 2. Clinical Utility vs. Statistical Metrics: Regarding the model performances, the Weighted Logistic Regression (WLR) is highlighted in the Abstract and Discussion for achieving the highest accuracy and specificity. However, as shown in Table 4, its recall is extremely low (0.070) on the test set. In a clinical or public health context, a predictive model that misses 93% of positive cases has limited practical utility. While the authors acknowledge this limitation in the conclusion, I recommend slightly adjusting the narrative—particularly in the Abstract—to avoid overstating the WLR's success based solely on accuracy. Emphasizing that models like Random Forest are arguably more valuable for actual public health deployment (due to their higher sensitivity/recall) would provide a more realistic assessment of their practical value.

**Author’s response:** Thank you for this insightful comment. We have revised the manuscript to present a more balanced interpretation of the model performance. Specifically, we modified the Abstract, Discussion, and Conclusion to avoid overstating the performance of the Weighted Logistic Regression model based primarily on its accuracy. We now explicitly acknowledge its poor recall and the resulting limitation in identifying hypertensive individuals. In addition, we emphasize that the Random Forest model, with its substantially higher recall, is likely to be more suitable for public health (Page 2, lines 26 - 36; page 19, lines 386, 388; page 21, lines 438 - 441).

**Reviewer #3’s Comments:** I asked earlier about accounting for the survey design, and the response ("stratified split by hypertension status") doesn't really answer it. Stratifying by the outcome isn't survey-aware and does nothing about sampling weights or clustering. Please say clearly whether DHS weights went into any of the models, and justify it either way.

**Author’s response:** Thank you for this important clarification. We agree that stratifying the train–test split by hypertension status does not account for the complex DHS survey design. In our analysis, the DHS sampling weights were used only in the weighted logistic regression, which is the baseline survey-weighted regression model. DHS sampling weights were not used in the training of the random forest, XGBoost, LightGBM, MLP, and TabNet models. We made this distinction because the use of the ML and DL models in this study was exploratory, with the primary objective being to compare the predictive performance of various ML and DL algorithms with a survey-weighted logistic regression model.

We have revised the manuscript to state explicitly that DHS sampling weights were used only in the WLR model and were not incorporated into the other ML/DL models also mentioned in the limitation of the study section (Page 8, lines 161–165; page 20, lines 417 - 418).

**Reviewer #3’s Comments:** There's also a loose end here: the letter says the cross-validation grouped by survey clusters to avoid leakage, but the main 80/20 split (which the abstract's numbers come from) is only stratified by outcome. If the same cluster shows up in both train and test, those headline numbers are optimistic.

**Author’s response:** You are absolutely right that we could have considered k-fold cross-validation using only stratification by the outcome. However, our predictive class is imbalanced. Therefore, to assess the robustness of model performance, we used the widely adopted StratifiedGroupKFold cross-validation function from the scikit-learn package in Python. Additionally, we noted in the limitations section that DHS sampling weights were incorporated only into the WLR model and not into the other ML and DL models (Page 20, lines 417 – 418).

**Reviewer #3’s Comments:** A couple of numbers contradict your own tables. The CV text calls RF's mean AUC-ROC "≈ 0.44," but Table 5 says 0.748 (0.44 would be below chance). RF's F1 is given as 0.448 versus 0.443 in the table. Please correct these.

**Author’s response:** Thank you for identifying these inconsistencies. We carefully reviewed the reported performance metrics and corrected the typographical errors in the manuscript text (Page 17, Line 338).

**Reviewer #3’s Comments:** One framing point: WLR's 0.817 accuracy is basically the no-information rate (the majority class is 82%), and its recall is 0.070, so it's calling almost everyone non-hypertensive. Worth stating that directly and leaning on F1 and AUC-PR instead.

**Author’s response:** Thank you for this insightful observation. We have revised the manuscript to explicitly acknowledge the WLR model's very low recall (0.070), and this limitation is now also highlighted in the Abstract. We have also revised the Discussion to emphasize the F1-score and AUC-PR over accuracy for evaluating performance in this imbalanced dataset. Additionally, the Conclusion now notes that the RF model may be more suitable for public health applications because of its better ability to identify hypertensive individuals (Page 2, Lines 26 - 36; page 19, lines 386, 388; page 21, lines 438 - 441).

**Reviewer #3’s Comments:** Minor cleanup: the age bands don't match between Table 1 ("35–60", "60 and over") and the rest of the paper ("35–59", "60+"); the BMI "normal" range now overlaps the obese cutoff at 25; the Table 2 specificity description is wrong (formula is fine); and the revised file still carries some R0 leftovers, including the old MLP narrative and a duplicate "Strengths and Limitations" section.

**Author’s response:** Thank you for identifying these inconsistencies. We have carefully reviewed the manuscript and corrected the age group labels to ensure consistency throughout the paper, revised the BMI classification to remove the overlap in the normal and obese categories, corrected the description of specificity in Table 2, and removed the remaining R0 content, including the outdated MLP narrative and the duplicate "Strengths and Limitations" section (Page 6, Lines 124 – 125, 130; page 10-11, line 232; page 16, lines 310 - 313; page 20, lines 406 - 407).

We've incorporated all feedback from the reviewers, ensuring the revised manuscript aligns with PLOS ONE's high standards. We do believe the changes substantially enhance the research quality and contribution.

We reiterate our deep appreciation for their invaluable expertise and insights that greatly improved our work. We trust this revised version merits publication in PLOS ONE.

Thank you for your time and consideration.
