# Supplementary material for: Machine learning and deep learning–based prediction of hypertension and analysis of its major risk factors in Bangladesh
Source: PLoS One. 2026 Sep 17;21(9):e0358471. doi: 10.1371/journal.pone.0358471 (PMC13585235; doi:10.1371/journal.pone.0358471)
Supplement: Supplementary file 4 [file pone.0358471.s004.docx]

**Response to the Reviewers**

Dear Reviewers,

We would like to extend our heartfelt appreciation to the reviewers for generously devoting their time and offering insightful feedback on our manuscript titled "Machine and deep learning–based prediction of hypertension and analysis of its major risk factors in Bangladesh". We are grateful for the constructive comments provided, as they have enabled us to enhance the overall quality and coherence of our research. In light of the raised concerns, we have addressed each point in a comprehensive manner as outlined below:

**Reviewer #3’s Comments:** State explicitly that the 80/20 split is cluster-disjoint and note Table 5 mitigates this concern.

**Author’s response:** Thank you for raising this important concern. We carefully reviewed the original train–test splitting procedure and clarified that the primary 80/20 split was stratified by hypertension status but was not explicitly constrained to be cluster-disjoint. For cross-validation, we used StratifiedGroupKFold, which ensures that observations from the same cluster are not shared between the training and validation folds. We have stated this explicitly in the Methods, Results, and Discussion sections (Pages 7 - 8, Lines 158 – 161; Page 12, Lines 246 – 248; Page 17, Lines 333 – 335; Page 19, Lines 385 – 387).

**Reviewer #3’s Comments**: Add one sentence noting WLR's 0.817 accuracy is at or below the 81.96% majority class rate.

**Author’s response:** Thank you for this helpful observation. We have added a sentence to the Results section clarifying that the WLR accuracy is slightly below the majority-class rate (Page 15, Lines 304 - 306).

**Reviewer #3’s Comments:** Acknowledge that RF's higher recall reflects its more permissive operating point (SMOTE + balanced weights), not necessarily better discriminative ability, and soften the "superiority" framing.

**Author’s response:** Thank you for this important suggestion. In the Results and Discussion sections, we have clarified that the higher recall may reflect the more permissive operating point associated with SMOTE-based class balancing and balanced weighting, which favors the identification of minority-class observations. We have also softened the previous claims of superiority, particularly in the Abstract, and now emphasize that the Random Forest (RF) model achieved the highest recall and F1-score (Page 2, Line 30; Page 17, Lines 344 – 346; Page 19, Lines 390 - 393).

**Reviewer #3’s Comments:** Fix residual typos (Fig 2Fig 1, train/test value confusion, BMI boundary overlap).

**Author’s response:** Thank you for pointing out these remaining inconsistencies. We carefully reviewed the manuscript and corrected the residual figure-numbering typo, clarified the train/test dataset references to ensure that all reported performance values are correctly identified, and revised the BMI category definitions to use mutually exclusive boundaries. We also carefully reviewed the source codes (Page 6, Lines 124 – 125; Page 16, Lines 322; Page 19, Lines 387 – 388).

We've incorporated all feedback from the reviewers, ensuring the revised manuscript aligns with PLOS ONE's high standards. We do believe the changes substantially enhance the research quality and contribution.

We reiterate our deep appreciation for their invaluable expertise and insights that greatly improved our work. We trust this revised version merits publication in PLOS ONE.

Thank you for your time and consideration.
