# Supplementary material for: Machine learning and deep learning–based prediction of hypertension and analysis of its major risk factors in Bangladesh
Source: PLoS One. 2026 Sep 17;21(9):e0358471. doi: 10.1371/journal.pone.0358471 (PMC13585235; doi:10.1371/journal.pone.0358471)
Supplement: Peer review history

# Peer review: Decision Letter 0

**DOI:** 10.1371/journal.pone.0358471.r001

**Authors:** André Luis C Ramalho

12 Jan 2026

Dear Dr. Ali,

Thank you for submitting your manuscript to PLOS ONE. After careful consideration, we feel that it has merit but does not fully meet PLOS ONE’s publication criteria as it currently stands. Therefore, we invite you to submit a revised version of the manuscript that addresses the points raised during the review process.

Please submit your revised manuscript by Feb 26 2026 11:59PM. If you will need more time than this to complete your revisions, please reply to this message or contact the journal office at plosone@plos.org. When you're ready to submit your revision, log on to https://www.editorialmanager.com/pone/ and select the 'Submissions Needing Revision' folder to locate your manuscript file.

- A letter that responds to each point raised by the academic editor and reviewer(s). You should upload this letter as a separate file labeled 'Response to Reviewers'.
- A marked-up copy of your manuscript that highlights changes made to the original version. You should upload this as a separate file labeled 'Revised Manuscript with Track Changes'.
- An unmarked version of your revised paper without tracked changes. You should upload this as a separate file labeled 'Manuscript'.

If you would like to make changes to your financial disclosure, please include your updated statement in your cover letter. Guidelines for resubmitting your figure files are available below the reviewer comments at the end of this letter.

If applicable, we recommend that you deposit your laboratory protocols in protocols.io to enhance the reproducibility of your results. Protocols.io assigns your protocol its own identifier (DOI) so that it can be cited independently in the future. For instructions see: https://journals.plos.org/plosone/s/submission-guidelines#loc-laboratory-protocols. Additionally, PLOS ONE offers an option for publishing peer-reviewed Lab Protocol articles, which describe protocols hosted on protocols.io. Read more information on sharing protocols at https://plos.org/protocols?utm_medium=editorial-email&utm_source=authorletters&utm_campaign=protocols.

We look forward to receiving your revised manuscript.

Kind regards,

André Luis C Ramalho, PhD

Academic Editor

PLOS One

Journal requirements:

When submitting your revision, we need you to address these additional requirements.

1. Please ensure that your manuscript meets PLOS ONE's style requirements, including those for file naming. The PLOS ONE style templates can be found at

https://journals.plos.org/plosone/s/file?id=wjVg/PLOSOne_formatting_sample_main_body.pdf and

https://journals.plos.org/plosone/s/file?id=ba62/PLOSOne_formatting_sample_title_authors_affiliations.pdf

2. When completing the data availability statement of the submission form, you indicated that you will make your data available on acceptance. We strongly recommend all authors decide on a data sharing plan before acceptance, as the process can be lengthy and hold up publication timelines. Please note that, though access restrictions are acceptable now, your entire data will need to be made freely accessible if your manuscript is accepted for publication. This policy applies to all data except where public deposition would breach compliance with the protocol approved by your research ethics board. If you are unable to adhere to our open data policy, please kindly revise your statement to explain your reasoning and we will seek the editor's input on an exemption. Please be assured that, once you have provided your new statement, the assessment of your exemption will not hold up the peer review process.

If the reviewer comments include a recommendation to cite specific previously published works, please review and evaluate these publications to determine whether they are relevant and should be cited. There is no requirement to cite these works unless the editor has indicated otherwise.

Additional Editor Comments:

Dear Authors,

Thank you for submitting your manuscript entitled “Machine and deep learning–based prediction of hypertension and analysis of its major risk factors in Bangladesh” to PLOS ONE. Your study addresses an important public health topic and applies contemporary machine learning and deep learning approaches to nationally representative data, which aligns well with the journal’s scope and multidisciplinary readership.

After careful editorial assessment of the manuscript, including evaluation of its scientific rigor, methodological transparency, and potential contribution to the literature, I am writing to inform you of the editorial decision:

Decision: Major Revisions Required

Below, I provide a detailed editorial summary outlining the main strengths of the manuscript, followed by the key issues that must be addressed before the manuscript can be considered for further evaluation.

1. Overall Assessment

The manuscript presents a well-conducted cross-sectional analytical study using data from the 2022 Bangladesh Demographic and Health Survey (BDHS) to (i) estimate the prevalence of hypertension, (ii) examine associated socio-demographic and health-related factors, and (iii) develop predictive models using both machine learning (ML) and deep learning (DL) techniques.

The topic is relevant and timely, particularly for low- and middle-income countries facing a growing burden of noncommunicable diseases. The use of multiple ML/DL algorithms and comparison of their predictive performance represents a methodologically sound and potentially valuable contribution, although largely incremental relative to existing literature.

2. Key Strengths

Use of a large, nationally representative dataset with appropriate handling of the complex survey design in descriptive and inferential analyses.

Clear articulation of objectives and coherent structure across sections (Introduction, Methods, Results, Discussion).

Application of multiple ML and DL models, with transparent reporting of core performance metrics (accuracy, precision, AUC).

Appropriate acknowledgment of the main limitation related to the cross-sectional nature of the data.

Relevance to public health surveillance and population-level risk stratification in resource-limited settings.

3. Major Issues Requiring Revision

The following points must be addressed comprehensively in a revised version of the manuscript:

3.1. Reporting Standards and Methodological Transparency

The study design corresponds to an observational cross-sectional study, and therefore adherence to the STROBE reporting guidelines is expected.

Please include a completed STROBE checklist as a supplementary file and ensure that all relevant items are adequately addressed in the manuscript.

While the ML/DL modeling is described, key aspects related to model robustness require further clarification:

Explicit discussion of potential overfitting.

Clarification on whether any form of cross-validation (e.g., k-fold) was considered or why it was not applied.

Stronger justification for the choice of performance metrics and thresholds, particularly given the class imbalance typical of hypertension prevalence data.

3.2. Interpretation of Predictive Performance

The reported AUC values (approximately 0.73 at best) indicate moderate discriminatory performance.

The Discussion and Conclusions currently risk overstating the practical applicability of the models.

Please temper claims regarding the utility of DL models for real-world screening or decision-making, and clearly distinguish statistical performance from clinical or policy relevance.

Many of the most important predictors (e.g., age, BMI, education) are well-established risk factors.

Please clarify the added value of the ML/DL approach compared with traditional regression-based models beyond marginal gains in predictive accuracy.

3.3. Ethical Statement

The Ethics Statement currently reports “N/A.”

Although the study uses publicly available, anonymized secondary data, PLOS ONE requires a clear ethical justification, including:

Identification of the data source as publicly available.

A statement that the analysis involved no identifiable human subjects and therefore did not require institutional ethical approval, or confirmation of exemption where applicable.

3.4. Reproducibility and Open Science

In line with PLOS ONE’s commitment to transparency and reproducibility:

Please clarify whether the analysis code (R/Python scripts) can be shared, and if so, indicate how and where it will be made available (e.g., public repository).

If code sharing is not possible, provide a clear justification.

3.5. Discussion of Bias and Limitations

The manuscript would benefit from a more explicit discussion of:

Residual confounding and limitations inherent to self-reported or single-occasion measurements.

Potential algorithmic bias, particularly given socio-demographic and regional disparities.

The absence of behavioral variables (e.g., diet, physical activity, salt intake) and how this may affect predictive performance.

4. Minor but Important Points

Please ensure consistent terminology when referring to performance metrics (e.g., avoid interchangeably using “accuracy” and “precision” incorrectly).

Review the language in the Conclusions to ensure it remains fully supported by the results.

Carefully proofread the manuscript for minor grammatical and typographical issues.

5. Editorial Recommendation

The manuscript demonstrates scientific merit and relevance, but substantial revisions are required to strengthen methodological transparency, ethical reporting, and the interpretation of findings. Provided that the concerns outlined above are addressed thoroughly and convincingly, the manuscript may be suitable for reconsideration.

We invite you to submit a revised manuscript along with a detailed point-by-point response to each comment raised in this decision letter.

Thank you for your interest in PLOS ONE. We look forward to receiving your revised submission.

Sincerely,

Academic Editor

PLOS ONE

[Note: HTML markup is below. Please do not edit.]

Reviewers' comments:

Reviewer's Responses to Questions

**Comments to the Author**

1. Is the manuscript technically sound, and do the data support the conclusions?

Reviewer #1: No

**********

2. Has the statistical analysis been performed appropriately and rigorously?

Reviewer #1: No

**********

3. Have the authors made all data underlying the findings in their manuscript fully available?

The PLOS Data policy

Reviewer #1: Yes

**********

4. Is the manuscript presented in an intelligible fashion and written in standard English?

Reviewer #1: Yes

**********

Reviewer #1: Major compulsory revisions

Address class imbalance explicitly (e.g., via class weights in MLP/TabNet or SMOTE). Report full metrics (accuracy, precision, recall, specificity, F1, AUC-ROC, AUC-PR) in a table for all models on both train and test sets. Emphasize F1 or AUC-PR over accuracy in imbalanced contexts.

Implement k-fold cross-validation (stratified by hypertension status and survey clusters) for robust performance estimates. Report mean/SD across folds.

Include a baseline model (e.g., survey-weighted logistic regression) for comparison. If DL outperforms, quantify (e.g., via McNemar's test).

Specify how feature importance was computed (e.g., for MLP, use SHAP instead of unspecified method). Provide uncertainty (e.g., via bootstrapping).

Account for survey design in modeling (e.g., incorporate weights in loss functions or use survey-aware splits).

Tone down conclusions: Acknowledge modest AUC/F1 and limitations of cross-sectional data for causality. Avoid overclaiming DL "superiority" without baselines.

Expand limitations: Discuss lack of behavioral variables (e.g., diet, exercise) and potential overfitting. Suggest future longitudinal validation.

**********

what does this mean?). If published, this will include your full peer review and any attached files.

If you choose “no”, your identity will remain anonymous but your review may still be made public.

**Do you want your identity to be public for this peer review?**  For information about this choice, including consent withdrawal, please see our Privacy Policy

Reviewer #1: **Yes:** Ali Mirarab

**********

[NOTE: If reviewer comments were submitted as an attachment file, they will be attached to this email and accessible via the submission site. Please log into your account, locate the manuscript record, and check for the action link "View Attachments". If this link does not appear, there are no attachment files.]

To ensure your figures meet our technical requirements, please review our figure guidelines: https://journals.plos.org/plosone/s/figures

You may also use PLOS’s free figure tool, NAAS, to help you prepare publication quality figures: https://journals.plos.org/plosone/s/figures#loc-tools-for-figure-preparation.

NAAS will assess whether your figures meet our technical requirements by comparing each figure against our figure specifications.

Attachment Submitted filename: Revision Orientation PONE-D-25-55897.pdf

# Author comment: Author response to Decision Letter 1

**DOI:** 10.1371/journal.pone.0358471.r002

6 Apr 2026

Response to the Editor and Reviewer

Dear Editor and Reviewers,

We would like to extend our heartfelt appreciation to the academic editor and reviewers for generously devoting their time and offering insightful feedback on our manuscript titled "Machine and deep learning–based prediction of hypertension and analysis of its major risk factors in Bangladesh". We are grateful for the constructive comments provided, as they have enabled us to enhance the overall quality and coherence of our research. In light of the raised concerns, we have addressed each point in a comprehensive manner as outlined below:

3.1. Reporting Standards and Methodological Transparency

Editor comment: The study design corresponds to an observational cross-sectional study, and therefore adherence to the STROBE reporting guidelines is expected.

Author’s response: We thank the Editor for the helpful comments and suggestions. We have revised our manuscript and ensured full compliance with the STROBE reporting guidelines.

Editor comment: Please include a completed STROBE checklist as a supplementary file and ensure that all relevant items are adequately addressed in the manuscript.

Author’s response: We thank the Editor for this valuable suggestion. A completed STROBE checklist has now been included as supplementary file S1_file.pdf. All applicable STROBE items have been systematically addressed in the revised manuscript, including participant selection, variable definitions, handling of missing data, and statistical methods, and so on.

Editor comment: While the ML/DL modeling is described, key aspects related to model robustness require further clarification: Explicit discussion of potential overfitting.

Author’s response: We thank the Editor for this important comment. We have addressed potential overfitting in the subsection Overfitting in machine and deep learning model of Materials and Methods section (page: 11; lines: 232 - 243) and the Discussion (page: 18; lines: 370 - 375) sections.

Editor comment: Clarification on whether any form of cross-validation (e.g., k-fold) was considered or why it was not applied.

Author’s response: We thank the Editor for raising this point. We have used 5-fold and 10-fold cross-validation to validate our results. K-fold cross-validation has been explicitly discussed in the Materials and Methods section (page: 11; lines: 222 - 231) and in the Results section (page: 16; lines: 314 - 336).

Editor comment: Stronger justification for the choice of performance metrics and thresholds, particularly given the class imbalance typical of hypertension prevalence data.

Author’s response: We thank the Editor for this helpful suggestion. We have expanded the justification for the selected performance metrics in the Materials and Methods section (page: 10; lines: 206 - 221). We explain that metrics such as AUC-ROC, precision, recall, specificity, F1-score, and AUC-PR are more appropriate than accuracy alone for evaluating models trained on imbalanced outcome data such as hypertension prevalence.

3.2. Interpretation of Predictive Performance

Editor comment: The reported AUC values (approximately 0.73 at best) indicate moderate discriminatory performance.

Author’s response: We appreciate the Editor’s careful assessment. We agree with this assessment. We reanalyzed our data by applying SMOTE to address class imbalance, added a weighted logistic regression model, and expanded the set of performance metrics to include precision, recall, specificity, F1-score, AUC-PR, and AUC. Throughout the manuscript, we have avoided overstating the discriminatory performance of the model.

Editor comment: The Discussion and Conclusions currently risk overstating the practical applicability of the models.

Author’s response: We thank the Editor for this valuable observation. The Discussion and Conclusions have been revised to temper claims regarding practical applicability. The predictive performance is now described as moderate, and limitations related to real-world implementation are clearly acknowledged.

Editor comment: Please temper claims regarding the utility of DL models for real-world screening or decision-making, and clearly distinguish statistical performance from clinical or policy relevance.

Author’s response: We appreciate this important suggestion. We have clarified that the ML/DL models are exploratory in nature and are not intended for direct clinical screening or decision-making without further validation in the discussion section (page: 19; lines: 391 - 395).

Editor comment: Many of the most important predictors (e.g., age, BMI, education) are well-established risk factors.

Author’s response: We appreciate the Editor’s comment. We acknowledge that age, BMI, and education are well-established risk factors for hypertension. The discussion now explicitly situates these findings within the existing literature and cites previous studies to contextualize their importance in Discussion section (page:19; lines: 376 - 390).

Editor comment: Please clarify the added value of the ML/DL approach compared with traditional regression-based models beyond marginal gains in predictive accuracy.

Author’s response: We thank the Editor for this thoughtful comment. We have clarified that, in our study, the survey-weighted logistic regression model performed comparably or better on test data. The added value of ML/DL approaches is discussed in terms of their ability to capture nonlinear relationships rather than superior predictive accuracy, and claims of DL superiority have been avoided in the result and discussion section.

3.3. Ethical Statement

Editor comment: The Ethics Statement currently reports “N/A.”

Author’s response: We thank the Editor for highlighting this issue. We have revised the Ethics Statement to comply fully with PLOS ONE requirements

Editor comment: Although the study uses publicly available, anonymized secondary data, PLOS ONE requires a clear ethical justification, including:

Identification of the data source as publicly available.

A statement that the analysis involved no identifiable human subjects and therefore did not require institutional ethical approval, or confirmation of exemption where applicable.

Author’s response: We appreciate the Editor’s guidance. We have added a new sub-section titled 'Ethical Approval and Consent to Participate' (page 4: lines: 88–96). The revised statement clarifies that the study utilized publicly available, anonymized secondary data from the Bangladesh Demographic and Health Survey (BDHS). Since the research involved no identifiable human subjects, formal institutional ethical approval was not required.

3.4. Reproducibility and Open Science

Editor comment: In line with PLOS ONE’s commitment to transparency and reproducibility:

Please clarify whether the analysis code (R/Python scripts) can be shared, and if so, indicate how and where it will be made available (e.g., public repository).

If code sharing is not possible, provide a clear justification.

Author’s response: We thank the Editor for this important suggestion. We have created a new section titled “Data and Code Availability” (page: 5; lines: 97 - 102). This sub-section states that the analysis scripts used in this study are shared via a public repository, and the repository link has been provided.

3.5. Discussion of Bias and Limitations

Editor comment: The manuscript would benefit from a more explicit discussion of: Residual confounding and limitations inherent to self-reported or single-occasion measurements.

Author’s response: We thank the Editor for this valuable suggestion. We have created a new sub-section in the manuscript titled “Limitations of the Study” (page: 19; lines: 396 - 406). In this section, we note that the data are cross-sectional and self-reported, which may introduce residual confounding.

Editor comment: Potential algorithmic bias, particularly given socio-demographic and regional disparities.

Author’s response: We appreciate this insightful comment. In the sub-section “Limitations of the Study” (page: 19; lines: 396 - 406), we have added a discussion of potential algorithmic bias arising from socio-demographic and regional disparities.

Editor comment: The absence of behavioral variables (e.g., diet, physical activity, salt intake) and how this may affect predictive performance.

Author’s response: We thank the Editor for this helpful observation. In the sub-section “Limitations of the Study” (page: 19; lines: 396 - 406), we now explicitly acknowledge the absence of behavioral variables such as diet, physical activity, and salt intake as an important limitation and discuss how their exclusion may affect predictive performance.

4. Minor but Important Points

Editor comment: Please ensure consistent terminology when referring to performance metrics (e.g., avoid interchangeably using “accuracy” and “precision” incorrectly).

Author’s response: We thank the Editor for noting this issue. Throughout the manuscript, we have avoided the incorrect interchangeable use of terms such as accuracy and precision.

Editor comment: Review the language in the Conclusions to ensure it remains fully supported by the results.

Author’s response: We appreciate the Editor’s suggestion. The Conclusions have been revised to ensure that all statements are fully supported by the reported results and appropriately qualified.

Editor comment: Carefully proofread the manuscript for minor grammatical and typographical issues.

Author’s response: We thank the Editor for this suggestion. The manuscript has been carefully proofread, and minor grammatical and typographical issues have been corrected.

Reviewer #1: Address class imbalance explicitly (e.g., via class weights in MLP/TabNet or SMOTE). Report full metrics (accuracy, precision, recall, specificity, F1, AUC-ROC, AUC-PR) in a table for all models on both train and test sets. Emphasize F1 or AUC-PR over accuracy in imbalanced contexts.

Author’s response: We sincerely thank the Reviewer for his careful reading of the manuscript and his valuable comments. We have explicitly addressed class imbalance using SMOTE and provide performance metrics, including accuracy, precision, recall, specificity, F1-score, AUC-ROC, and AUC-PR for all models across both the training and test datasets. In the Materials and Methods section, we have added a sub-section on “Handling class imbalance” (page: 6; lines: 131 - 138) and updated the 'Performance Metrics' subsection (lines: 186 - 202) to further discuss the evaluation of imbalanced data.

Reviewer #1: Implement k-fold cross-validation (stratified by hypertension status and survey clusters) for robust performance estimates. Report mean/SD across folds.

Author’s response: We sincerely thank the Reviewer for this helpful suggestion. Stratified group k-fold cross-validation was used, with hypertension status guiding stratification and survey clusters treated as grouping units to prevent data leakage. We implemented 5-fold and 10-fold cross-validation and now report the mean values and standard deviations for all performance metrics across the folds. The K-fold cross-validation methodology is detailed in the Materials and Methods section (page: 10; lines: 206 - 221)., and the corresponding outcomes are presented in the Results section (page: 16; lines: 314 - 336).

Reviewer #1: Include a baseline model (e.g., survey-weighted logistic regression) for comparison. If DL outperforms, quantify (e.g., via McNemar's test).

Author’s response: We thank the Reviewer for this valuable suggestion. We have included a survey-weighted logistic regression (WLR) model as a baseline. The model is described in the Materials and Methods section (page: 7; lines: 153 - 163), and its findings are presented in the Results section.

Reviewer #1: Specify how feature importance was computed (e.g., for MLP, use SHAP instead of unspecified method). Provide uncertainty (e.g., via bootstrapping).

Author’s response: We appreciate the Reviewer’s suggestion. Feature importance was assessed using SHAP analysis, and the corresponding results are presented in the Results section (page 17; lines: 337 - 350).

Reviewer #1: Account for survey design in modeling (e.g., incorporate weights in loss functions or use survey-aware splits).

Author’s response: We thank the Reviewer for raising this important point. We have employed a stratified train–test split based on hypertension status to ensure that the class distribution was preserved across both subsets.

Reviewer #1: Tone down conclusions: Acknowledge modest AUC/F1 and limitations of cross-sectional data for causality. Avoid overclaiming DL "superiority" without baselines.

Author’s response: We sincerely thank the Reviewer for this helpful recommendation. We have toned down the Conclusions (page: 20; lines: 407 - 427), acknowledged the modest AUC and F1 values, acknowledged no superiority of a single model, and avoided claims of DL superiority.

Reviewer #1: Expand limitations: Discuss lack of behavioral variables (e.g., diet, exercise) and potential overfitting. Suggest future longitudinal validation.

Author’s response: We appreciate the Reviewer’s valuable suggestion. We have expanded the Limitations section (page: 19; lines: 396 - 406) to address the cross-sectional design of the study, the absence of behavioral data, potential risks of overfitting, and the need for longitudinal studies.

We've incorporated all feedback from the editor and reviewers, ensuring the revised manuscript aligns with PLOS ONE's high standards. We do believe the changes substantially enhance the research quality and contribution.

We reiterate our deep appreciation for their invaluable expertise and insights that greatly improved our work. We trust this revised version merits publication in PLOS ONE.

Thank you for your time and consideration.

Attachment Submitted filename: Response to Reviewers.docx

# Peer review: Decision Letter 1

**DOI:** 10.1371/journal.pone.0358471.r003

**Authors:** André Luis C Ramalho

21 Jul 2026

Dear Dr. Ali,

Thank you for submitting your manuscript to PLOS ONE. After careful consideration, we feel that it has merit but does not fully meet PLOS ONE’s publication criteria as it currently stands. Therefore, we invite you to submit a revised version of the manuscript that addresses the points raised during the review process.

Please submit your revised manuscript by Sep 04 2026 11:59PM. If you will need more time than this to complete your revisions, please reply to this message or contact the journal office at plosone@plos.org. When you're ready to submit your revision, log on to https://www.editorialmanager.com/pone/ and select the 'Submissions Needing Revision' folder to locate your manuscript file.

- A letter that responds to each point raised by the academic editor and reviewer(s). You should upload this letter as a separate file labeled 'Response to Reviewers'.
- A marked-up copy of your manuscript that highlights changes made to the original version. You should upload this as a separate file labeled 'Revised Manuscript with Track Changes'.
- An unmarked version of your revised paper without tracked changes. You should upload this as a separate file labeled 'Manuscript'.

If you would like to make changes to your financial disclosure, please include your updated statement in your cover letter. Guidelines for resubmitting your figure files are available below the reviewer comments at the end of this letter.

If applicable, we recommend that you deposit your laboratory protocols in protocols.io to enhance the reproducibility of your results. Protocols.io assigns your protocol its own identifier (DOI) so that it can be cited independently in the future. For instructions see: https://journals.plos.org/plosone/s/submission-guidelines#loc-laboratory-protocols. Additionally, PLOS ONE offers an option for publishing peer-reviewed Lab Protocol articles, which describe protocols hosted on protocols.io. Read more information on sharing protocols at https://plos.org/protocols?utm_medium=editorial-email&utm_source=authorletters&utm_campaign=protocols.

As the corresponding author, your ORCID iD is verified in the submission system and will appear in the published article. PLOS supports the use of ORCID, and we encourage all coauthors to register for an ORCID iD and use it as well. Please encourage your coauthors to verify their ORCID iD within the submission system before final acceptance, as unverified ORCID iDs will not appear in the published article. *Only*  the individual author can complete the verification step; PLOS staff *cannot*  verify ORCID iDs on behalf of authors.

We look forward to receiving your revised manuscript.

Kind regards,

André Luis C Ramalho, PhD

Academic Editor

PLOS One

Journal Requirements:

If the reviewer comments include a recommendation to cite specific previously published works, please review and evaluate these publications to determine whether they are relevant and should be cited. There is no requirement to cite these works unless the editor has indicated otherwise.

Please review your reference list to ensure that it is complete and correct. If you have cited papers that have been retracted, please include the rationale for doing so in the manuscript text, or remove these references and replace them with relevant current references. Any changes to the reference list should be mentioned in the rebuttal letter that accompanies your revised manuscript. If you need to cite a retracted article, indicate the article’s retracted status in the References list and also include a citation and full reference for the retraction notice.

[Note: HTML markup is below. Please do not edit.]

Reviewers' comments:

Reviewer's Responses to Questions

**Comments to the Author**

Reviewer #2: (No Response)

Reviewer #3: (No Response)

**********

2. Is the manuscript technically sound, and do the data support the conclusions?

Reviewer #2: Yes

Reviewer #3: Partly

**********

3. Has the statistical analysis been performed appropriately and rigorously?

Reviewer #2: Yes

Reviewer #3: Yes

**********

4. Have the authors made all data underlying the findings in their manuscript fully available?

The PLOS Data policy

Reviewer #2: Yes

Reviewer #3: Yes

**********

5. Is the manuscript presented in an intelligible fashion and written in standard English?

Reviewer #2: Yes

Reviewer #3: Yes

**********

Reviewer #2: The authors have done a commendable job revising the manuscript, and the methodology is generally robust. However, I have two minor points that I believe would further strengthen the paper's narrative and clinical applicability:

1. Contextualization of Local Impact:

While the introduction effectively outlines the global burden of hypertension, it lacks specific data regarding its impact within Bangladesh itself. The transition from the global context to the local scenario would be significantly stronger if the authors included local statistics on the consequences of the disease (e.g., mortality rates, morbidity, or specific economic burden in Bangladesh), rather than just stating that its prevalence is rising.

2. Clinical Utility vs. Statistical Metrics:

Regarding the model performances, the Weighted Logistic Regression (WLR) is highlighted in the Abstract and Discussion for achieving the highest accuracy and specificity. However, as shown in Table 4, its recall is extremely low (0.070) on the test set. In a clinical or public health context, a predictive model that misses 93% of positive cases has limited practical utility. While the authors acknowledge this limitation in the conclusion, I recommend slightly adjusting the narrative—particularly in the Abstract—to avoid overstating the WLR's success based solely on accuracy. Emphasizing that models like Random Forest are arguably more valuable for actual public health deployment (due to their higher sensitivity/recall) would provide a more realistic assessment of their practical value.

Reviewer #3: This is a solid revision. The authors added SMOTE, a full train/test metrics table, a logistic regression baseline, k-fold cross-validation, and SHAP importance, and they walked back the earlier "deep learning wins" claim. The paper now says plainly that the classical models do as well or better and the deep models overfit. That's the honest read, and I appreciate it. I recommend minor revision, with a few things to fix.

The main one: I asked earlier about accounting for the survey design, and the response ("stratified split by hypertension status") doesn't really answer it. Stratifying by the outcome isn't survey-aware and does nothing about sampling weights or clustering. Please say clearly whether DHS weights went into any of the models, and justify it either way. There's also a loose end here: the letter says the cross-validation grouped by survey clusters to avoid leakage, but the main 80/20 split (which the abstract's numbers come from) is only stratified by outcome. If the same cluster shows up in both train and test, those headline numbers are optimistic.

A couple of numbers contradict your own tables. The CV text calls RF's mean AUC-ROC "≈ 0.44," but Table 5 says 0.748 (0.44 would be below chance). RF's F1 is given as 0.448 versus 0.443 in the table. Please correct these.

One framing point: WLR's 0.817 accuracy is basically the no-information rate (the majority class is 82%), and its recall is 0.070, so it's calling almost everyone non-hypertensive. Worth stating that directly and leaning on F1 and AUC-PR instead.

Minor cleanup: the age bands don't match between Table 1 ("35–60", "60 and over") and the rest of the paper ("35–59", "60+"); the BMI "normal" range now overlaps the obese cutoff at 25; the Table 2 specificity description is wrong (formula is fine); and the revised file still carries some R0 leftovers, including the old MLP narrative and a duplicate "Strengths and Limitations" section.

Nice work overall.

**********

what does this mean?). If published, this will include your full peer review and any attached files.

If you choose “no”, your identity will remain anonymous but your review may still be made public.

**Do you want your identity to be public for this peer review?**  For information about this choice, including consent withdrawal, please see our Privacy Policy

Reviewer #2: **Yes:** Luiza Volfa de Souza

Reviewer #3: **Yes:** Md Abubakkar

**********

[NOTE: If reviewer comments were submitted as an attachment file, they will be attached to this email and accessible via the submission site. Please log into your account, locate the manuscript record, and check for the action link "View Attachments". If this link does not appear, there are no attachment files.]

To ensure your figures meet our technical requirements, please review our figure guidelines: https://journals.plos.org/plosone/s/figures

You may also use PLOS’s free figure tool, NAAS, to help you prepare publication quality figures: https://journals.plos.org/plosone/s/figures#loc-tools-for-figure-preparation.

NAAS will assess whether your figures meet our technical requirements by comparing each figure against our figure specifications.

# Author comment: Author response to Decision Letter 2

**DOI:** 10.1371/journal.pone.0358471.r004

30 Jul 2026

Response to the Reviewers

Dear Reviewers,

We would like to extend our heartfelt appreciation to the reviewers for generously devoting their time and offering insightful feedback on our manuscript titled "Machine and deep learning–based prediction of hypertension and analysis of its major risk factors in Bangladesh". We are grateful for the constructive comments provided, as they have enabled us to enhance the overall quality and coherence of our research. In light of the raised concerns, we have addressed each point in a comprehensive manner as outlined below:

Reviewer #2’s Comments: 1. Contextualization of Local Impact: While the introduction effectively outlines the global burden of hypertension, it lacks specific data regarding its impact within Bangladesh itself. The transition from the global context to the local scenario would be significantly stronger if the authors included local statistics on the consequences of the disease (e.g., mortality rates, morbidity, or specific economic burden in Bangladesh), rather than just stating that its prevalence is rising.

Author’s response: Thank you for this valuable suggestion. We agree that the original introduction did not sufficiently contextualize the burden of hypertension within Bangladesh. In response, we have expanded the introduction to include Bangladesh-specific evidence on the health and economic impact of hypertension (Page 3, Lines 48 - 54).

Reviewer #2’s Comments: 2. Clinical Utility vs. Statistical Metrics: Regarding the model performances, the Weighted Logistic Regression (WLR) is highlighted in the Abstract and Discussion for achieving the highest accuracy and specificity. However, as shown in Table 4, its recall is extremely low (0.070) on the test set. In a clinical or public health context, a predictive model that misses 93% of positive cases has limited practical utility. While the authors acknowledge this limitation in the conclusion, I recommend slightly adjusting the narrative—particularly in the Abstract—to avoid overstating the WLR's success based solely on accuracy. Emphasizing that models like Random Forest are arguably more valuable for actual public health deployment (due to their higher sensitivity/recall) would provide a more realistic assessment of their practical value.

Author’s response: Thank you for this insightful comment. We have revised the manuscript to present a more balanced interpretation of the model performance. Specifically, we modified the Abstract, Discussion, and Conclusion to avoid overstating the performance of the Weighted Logistic Regression model based primarily on its accuracy. We now explicitly acknowledge its poor recall and the resulting limitation in identifying hypertensive individuals. In addition, we emphasize that the Random Forest model, with its substantially higher recall, is likely to be more suitable for public health (Page 2, lines 26 - 36; page 19, lines 386, 388; page 21, lines 438 - 441).

Reviewer #3’s Comments: I asked earlier about accounting for the survey design, and the response ("stratified split by hypertension status") doesn't really answer it. Stratifying by the outcome isn't survey-aware and does nothing about sampling weights or clustering. Please say clearly whether DHS weights went into any of the models, and justify it either way.

Author’s response: Thank you for this important clarification. We agree that stratifying the train–test split by hypertension status does not account for the complex DHS survey design. In our analysis, the DHS sampling weights were used only in the weighted logistic regression, which is the baseline survey-weighted regression model. DHS sampling weights were not used in the training of the random forest, XGBoost, LightGBM, MLP, and TabNet models. We made this distinction because the use of the ML and DL models in this study was exploratory, with the primary objective being to compare the predictive performance of various ML and DL algorithms with a survey-weighted logistic regression model.

We have revised the manuscript to state explicitly that DHS sampling weights were used only in the WLR model and were not incorporated into the other ML/DL models also mentioned in the limitation of the study section (Page 8, lines 161–165; page 20, lines 417 - 418).

Reviewer #3’s Comments: There's also a loose end here: the letter says the cross-validation grouped by survey clusters to avoid leakage, but the main 80/20 split (which the abstract's numbers come from) is only stratified by outcome. If the same cluster shows up in both train and test, those headline numbers are optimistic.

Author’s response: You are absolutely right that we could have considered k-fold cross-validation using only stratification by the outcome. However, our predictive class is imbalanced. Therefore, to assess the robustness of model performance, we used the widely adopted StratifiedGroupKFold cross-validation function from the scikit-learn package in Python. Additionally, we noted in the limitations section that DHS sampling weights were incorporated only into the WLR model and not into the other ML and DL models (Page 20, lines 417 – 418).

Reviewer #3’s Comments: A couple of numbers contradict your own tables. The CV text calls RF's mean AUC-ROC "≈ 0.44," but Table 5 says 0.748 (0.44 would be below chance). RF's F1 is given as 0.448 versus 0.443 in the table. Please correct these.

Author’s response: Thank you for identifying these inconsistencies. We carefully reviewed the reported performance metrics and corrected the typographical errors in the manuscript text (Page 17, Line 338).

Reviewer #3’s Comments: One framing point: WLR's 0.817 accuracy is basically the no-information rate (the majority class is 82%), and its recall is 0.070, so it's calling almost everyone non-hypertensive. Worth stating that directly and leaning on F1 and AUC-PR instead.

Author’s response: Thank you for this insightful observation. We have revised the manuscript to explicitly acknowledge the WLR model's very low recall (0.070), and this limitation is now also highlighted in the Abstract. We have also revised the Discussion to emphasize the F1-score and AUC-PR over accuracy for evaluating performance in this imbalanced dataset. Additionally, the Conclusion now notes that the RF model may be more suitable for public health applications because of its better ability to identify hypertensive individuals (Page 2, Lines 26 - 36; page 19, lines 386, 388; page 21, lines 438 - 441).

Reviewer #3’s Comments: Minor cleanup: the age bands don't match between Table 1 ("35–60", "60 and over") and the rest of the paper ("35–59", "60+"); the BMI "normal" range now overlaps the obese cutoff at 25; the Table 2 specificity description is wrong (formula is fine); and the revised file still carries some R0 leftovers, including the old MLP narrative and a duplicate "Strengths and Limitations" section.

Author’s response: Thank you for identifying these inconsistencies. We have carefully reviewed the manuscript and corrected the age group labels to ensure consistency throughout the paper, revised the BMI classification to remove the overlap in the normal and obese categories, corrected the description of specificity in Table 2, and removed the remaining R0 content, including the outdated MLP narrative and the duplicate "Strengths and Limitations" section (Page 6, Lines 124 – 125, 130; page 10-11, line 232; page 16, lines 310 - 313; page 20, lines 406 - 407).

We've incorporated all feedback from the reviewers, ensuring the revised manuscript aligns with PLOS ONE's high standards. We do believe the changes substantially enhance the research quality and contribution.

We reiterate our deep appreciation for their invaluable expertise and insights that greatly improved our work. We trust this revised version merits publication in PLOS ONE.

Thank you for your time and consideration.

Attachment Submitted filename: Response_to_Reviewers_auresp_2.docx

# Peer review: Decision Letter 2

**DOI:** 10.1371/journal.pone.0358471.r005

**Authors:** André Luis C Ramalho

24 Aug 2026

Dear Dr. Ali,

Thank you for submitting your manuscript to PLOS One. After careful consideration, we feel that it has merit but does not fully meet PLOS One’s publication criteria as it currently stands. Therefore, we invite you to submit a revised version of the manuscript that addresses the points raised during the review process.

Please submit your revised manuscript by Oct 08 2026 11:59PM. If you will need more time than this to complete your revisions, please reply to this message or contact the journal office at plosone@plos.org. When you're ready to submit your revision, log on to https://www.editorialmanager.com/pone/ and select the 'Submissions Needing Revision' folder to locate your manuscript file.

- A letter that responds to each point raised by the academic editor and reviewer(s). You should upload this letter as a separate file labeled 'Response to Reviewers'.
- A marked-up copy of your manuscript that highlights changes made to the original version. You should upload this as a separate file labeled 'Revised Manuscript with Track Changes'.
- An unmarked version of your revised paper without tracked changes. You should upload this as a separate file labeled 'Manuscript'.

If you would like to make changes to your financial disclosure, please include your updated statement in your cover letter. Guidelines for resubmitting your figure files are available below the reviewer comments at the end of this letter.

If applicable, we recommend that you deposit your laboratory protocols in protocols.io to enhance the reproducibility of your results. Protocols.io assigns your protocol its own identifier (DOI) so that it can be cited independently in the future. For instructions see: https://journals.plos.org/plosone/s/submission-guidelines#loc-laboratory-protocols. Additionally, PLOS One offers an option for publishing peer-reviewed Lab Protocol articles, which describe protocols hosted on protocols.io. Read more information on sharing protocols at https://plos.org/protocols?utm_medium=editorial-email&utm_source=authorletters&utm_campaign=protocols.

As the corresponding author, your ORCID iD is verified in the submission system and will appear in the published article. PLOS supports the use of ORCID, and we encourage all coauthors to register for an ORCID iD and use it as well. Please encourage your coauthors to verify their ORCID iD within the submission system before final acceptance, as unverified ORCID iDs will not appear in the published article. *Only*  the individual author can complete the verification step; PLOS staff *cannot*  verify ORCID iDs on behalf of authors.

We look forward to receiving your revised manuscript.

Kind regards,

André Luis C Ramalho, PhD

Academic Editor

PLOS One

**Journal Requirements:**

1. If the reviewer comments include a recommendation to cite specific previously published works, please review and evaluate these publications to determine whether they are relevant and should be cited. There is no requirement to cite these works unless the editor has indicated otherwise.

2. Please review your reference list to ensure that it is complete and correct. If you have cited papers that have been retracted, please include the rationale for doing so in the manuscript text, or remove these references and replace them with relevant current references. Any changes to the reference list should be mentioned in the rebuttal letter that accompanies your revised manuscript. If you need to cite a retracted article, indicate the article’s retracted status in the References list and also include a citation and full reference for the retraction notice.

[Note: HTML markup is below. Please do not edit.]

Reviewers' comments:

Reviewer's Responses to Questions

**Comments to the Author**

Reviewer #2: All comments have been addressed

Reviewer #3: (No Response)

**********

2. Is the manuscript technically sound, and do the data support the conclusions?

Reviewer #2: Yes

Reviewer #3: Yes

**********

3. Has the statistical analysis been performed appropriately and rigorously?

Reviewer #2: Yes

Reviewer #3: Yes

**********

4. Have the authors made all data underlying the findings in their manuscript fully available?

The PLOS Data policy

Reviewer #2: Yes

Reviewer #3: Yes

**********

5. Is the manuscript presented in an intelligible fashion and written in standard English?

Reviewer #2: Yes

Reviewer #3: Yes

**********

Reviewer #2: I have reviewed the current version of the manuscript. The authors have adequately addressed all previous comments and concerns. The methodology is sound, and the conclusions are well-supported by the data presented. I have no further recommendations and support the acceptance of this manuscript for publication in its current form.

Reviewer #3: The manuscript adequately addresses R1 concerns on sampling weights, numeric errors, and cross-validation rationale. Table 5's close agreement with test-set results confirms cluster overlap does not materially inflate performance. Four minor fixes remain: (1) state explicitly that the 80/20 split is cluster-disjoint and note Table 5 mitigates this concern, (2) add one sentence noting WLR's 0.817 accuracy is at or below the 81.96% majority class rate, (3) acknowledge that RF's higher recall reflects its more permissive operating point (SMOTE + balanced weights), not necessarily better discriminative ability, and soften the "superiority" framing, and (4) fix residual typos (Fig 2Fig 1, train/test value confusion, BMI boundary overlap). None requires new analysis.

**********

what does this mean?). If published, this will include your full peer review and any attached files.

If you choose “no”, your identity will remain anonymous but your review may still be made public.

**Do you want your identity to be public for this peer review?**  For information about this choice, including consent withdrawal, please see our Privacy Policy

Reviewer #2: **Yes:** Luiza Volfa

Reviewer #3: **Yes:** Md Abubakkar

**********

[NOTE: If reviewer comments were submitted as an attachment file, they will be attached to this email and accessible via the submission site. Please log into your account, locate the manuscript record, and check for the action link "View Attachments". If this link does not appear, there are no attachment files.]

To ensure your figures meet our technical requirements, please review our figure guidelines: https://journals.plos.org/plosone/s/figures

You may also use PLOS’s free figure tool, NAAS, to help you prepare publication quality figures: https://journals.plos.org/plosone/s/figures#loc-tools-for-figure-preparation.

NAAS will assess whether your figures meet our technical requirements by comparing each figure against our figure specifications.

# Author comment: Author response to Decision Letter 3

**DOI:** 10.1371/journal.pone.0358471.r006

27 Aug 2026

Response to the Reviewers

Dear Reviewers,

We would like to extend our heartfelt appreciation to the reviewers for generously devoting their time and offering insightful feedback on our manuscript titled "Machine and deep learning–based prediction of hypertension and analysis of its major risk factors in Bangladesh". We are grateful for the constructive comments provided, as they have enabled us to enhance the overall quality and coherence of our research. In light of the raised concerns, we have addressed each point in a comprehensive manner as outlined below:

Reviewer #3’s Comments: State explicitly that the 80/20 split is cluster-disjoint and note Table 5 mitigates this concern.

Author’s response: Thank you for raising this important concern. We carefully reviewed the original train–test splitting procedure and clarified that the primary 80/20 split was stratified by hypertension status but was not explicitly constrained to be cluster-disjoint. For cross-validation, we used StratifiedGroupKFold, which ensures that observations from the same cluster are not shared between the training and validation folds. We have stated this explicitly in the Methods, Results, and Discussion sections (Pages 7 - 8, Lines 158 – 161; Page 12, Lines 246 – 248; Page 17, Lines 333 – 335; Page 19, Lines 385 – 387).

Reviewer #3’s Comments: Add one sentence noting WLR's 0.817 accuracy is at or below the 81.96% majority class rate.

Author’s response: Thank you for this helpful observation. We have added a sentence to the Results section clarifying that the WLR accuracy is slightly below the majority-class rate (Page 15, Lines 304 - 306).

Reviewer #3’s Comments: Acknowledge that RF's higher recall reflects its more permissive operating point (SMOTE + balanced weights), not necessarily better discriminative ability, and soften the "superiority" framing.

Author’s response: Thank you for this important suggestion. In the Results and Discussion sections, we have clarified that the higher recall may reflect the more permissive operating point associated with SMOTE-based class balancing and balanced weighting, which favors the identification of minority-class observations. We have also softened the previous claims of superiority, particularly in the Abstract, and now emphasize that the Random Forest (RF) model achieved the highest recall and F1-score (Page 2, Line 30; Page 17, Lines 344 – 346; Page 19, Lines 390 - 393).

Reviewer #3’s Comments: Fix residual typos (Fig 2Fig 1, train/test value confusion, BMI boundary overlap).

Author’s response: Thank you for pointing out these remaining inconsistencies. We carefully reviewed the manuscript and corrected the residual figure-numbering typo, clarified the train/test dataset references to ensure that all reported performance values are correctly identified, and revised the BMI category definitions to use mutually exclusive boundaries. We also carefully reviewed the source codes (Page 6, Lines 124 – 125; Page 16, Lines 322; Page 19, Lines 387 – 388).

We've incorporated all feedback from the reviewers, ensuring the revised manuscript aligns with PLOS ONE's high standards. We do believe the changes substantially enhance the research quality and contribution.

We reiterate our deep appreciation for their invaluable expertise and insights that greatly improved our work. We trust this revised version merits publication in PLOS ONE.

Thank you for your time and consideration.

Attachment Submitted filename: Response_to_Reviewers_auresp_3.docx

# Editor report: Decision Letter 3

**DOI:** 10.1371/journal.pone.0358471.r007

**Authors:** André Luis C Ramalho

1 Sep 2026

Machine learning and deep learning–based prediction of hypertension and analysis of its major risk factors in Bangladesh

PONE-D-25-55897R3

Dear Dr. Ali,

We’re pleased to inform you that your manuscript has been judged scientifically suitable for publication and will be formally accepted for publication once it meets all outstanding technical requirements.

Within one week, you’ll receive an e-mail detailing the required amendments. When these have been addressed, you’ll receive a formal acceptance letter and your manuscript will be scheduled for publication.

An invoice will be generated when your article is formally accepted. Please note, if your institution has a publishing partnership with PLOS and your article meets the relevant criteria, all or part of your publication costs will be covered. Please make sure your user information is up-to-date by logging into Editorial Manager at Editorial Manager® and clicking the ‘Update My Information' link at the top of the page. For questions related to billing, please contact billing support.

If your institution or institutions have a press office, please notify them about your upcoming paper to help maximize its impact. If they’ll be preparing press materials, please inform our press team as soon as possible -- no later than 48 hours after receiving the formal acceptance. Your manuscript will remain under strict press embargo until 2 pm Eastern Time on the date of publication. For more information, please contact onepress@plos.org.

Kind regards,

André Luis C Ramalho, PhD

Academic Editor

PLOS One

Reviewers' comments:

The authors have satisfactorily addressed the points raised during the previous round of review, and I have no further substantive concerns regarding the manuscript. The revised version is suitable for publication in its current form.

# Editor report: Acceptance letter

**DOI:** 10.1371/journal.pone.0358471.r008

**Authors:** André Luis C Ramalho

PONE-D-25-55897R3

PLOS One

Dear Dr. Ali,

I'm pleased to inform you that your manuscript has been deemed suitable for publication in PLOS One. Congratulations! Your manuscript is now being handed over to our production team.

At this stage, our production department will prepare your paper for publication. This includes ensuring the following:

* All references, tables, and figures are properly cited

* All relevant supporting information is included in the manuscript submission,

* There are no issues that prevent the paper from being properly typeset

You will receive further instructions from the production team, including instructions on how to review your proof when it is ready. Please keep in mind that we are working through a large volume of accepted articles, so please give us a few days to review your paper and let you know the next and final steps.

Lastly, if your institution or institutions have a press office, please let them know about your upcoming paper now to help maximize its impact. If they'll be preparing press materials, please inform our press team within the next 48 hours. Your manuscript will remain under strict press embargo until 2 pm Eastern Time on the date of publication. For more information, please contact onepress@plos.org.

You will receive an invoice from PLOS for your publication fee after your manuscript has reached the completed accept phase. If you receive an email requesting payment before acceptance or for any other service, this may be a phishing scheme. Learn how to identify phishing emails and protect your accounts at https://explore.plos.org/phishing.

If we can help with anything else, please email us at customercare@plos.org.

Thank you for submitting your work to PLOS One and supporting open access.

Kind regards,

PLOS One Editorial Office Staff

on behalf of

Prof. Dr. André Luis C Ramalho

Academic Editor

PLOS One
